# Low molecular weight heparin promotes the PPAR pathway by protecting the glycocalyx of cells to delay the progression of diabetic nephropathy

**DOI:** 10.1016/j.jbc.2024.107493

**Published:** 2024-06-24

**Authors:** Bin Zhang, Changkai Bu, Qingchi Wang, Qingqing Chen, Deling Shi, Hongyan Qiu, Zhangjie Wang, Jian Liu, Zhe Wang, Qunye Zhang, Lianli Chi

**Affiliations:** 1 National Glycoengineering Research Center, Shandong University, Qingdao, Shandong, China; 2 Department of Endocrinology and Metabolism, Affiliated Hospital of Weifang Medical University, Weifang, Shandong, China; 3 Division of Chemical Biology and Medicinal Chemistry, Eshelman School of Pharmacy, University of North Carolina, Chapel Hill, North Carolina, USA; 4 Department of Geriatrics, Shandong Provincial Hospital Affiliated to Shandong First Medical University, Jinan, China; 5 Department of Endocrinology & Geriatrics, Shandong Provincial Hospital, Shandong University, Jinan, China; 6 National Key Laboratory for Innovation and Transformation of Luobing Theory, Jinan, Shandong, China; 7 The Key Laboratory of Cardiovascular Remodeling and Function Research, Chinese Ministry of Education, Chinese National Health Commission and Chinese Academy of Medical Sciences, Jinan, Shandong, China; 8 Department of Cardiology, Qilu Hospital of Shandong University, Jinan, Shandong, China

**Keywords:** diabetic nephropathy, low molecular weight heparin, heparan sulfate, FABP1, endocytosis, PPAR pathway

## Abstract

Diabetic nephropathy (DN) is one of the most important comorbidities for diabetic patients, which is the main factor leading to end-stage renal disease. Heparin analogs can delay the progression of DN, but the mechanism is not fully understood. In this study, we found that low molecular weight heparin therapy significantly upregulated some downstream proteins of the peroxisome proliferator–activated receptor (PPAR) signaling pathway by label-free quantification of the mouse kidney proteome. Through cell model verification, low molecular weight heparin can protect the heparan sulfate of renal tubular epithelial cells from being degraded by heparanase that is highly expressed in a high-glucose environment, enhance the endocytic recruitment of fatty acid–binding protein 1, a coactivator of the PPAR pathway, and then regulate the activation level of intracellular PPAR. In addition, we have elucidated for the first time the molecular mechanism of heparan sulfate and fatty acid–binding protein 1 interaction. These findings provide new insights into understanding the role of heparin in the pathogenesis of DN and developing corresponding treatments.

Diabetic nephropathy (DN) is a major microvascular complication of diabetes, occurring in approximately 30% of patients with type 1 diabetes mellitus and approximately 40% of patients with type 2 diabetes mellitus. DN is characterized by persistent proteinuria, elevated arterial blood pressure and a persistent decrease in glomerular filtration rate and is the leading cause of morbidity and mortality in diabetics, leading not only to end-stage renal disease but also an increase in cardiovascular adverse events (1, 2). At present, the focus of clinical treatment of DN is mainly antihypertensive and antiproteinuria, but the specific treatment of DN has not been determined (3). Therefore, accelerating the development of novel therapeutics is critical to improving the current disease situation.

Proteoglycans (PGs) are components of the glycocalyx in the extracellular matrix, and the glycosaminoglycan (GAG) glycan chains on PGs (particularly heparan sulfate (HS) PGs, HSPGs) play a key role in cellular and tissue homeostasis by interacting with a variety of proteins and regulating various processes such as proliferation, differentiation, angiogenesis and inflammation, thereby participating in and intervening in a variety of human diseases, including DN (4, 5). Several studies have shown that long-term hyperglycemia in diabetic patients induces the alteration and destruction of HS molecular structure on HSPG through multiple pathways and plays an important role in DN (6, 7). The glomerular basement membrane (GBM) is an ordered network composed of proteins and HSPGs, on which the HS determines its ionic charge permeability characteristics, and the upregulation of heparanase expression in diabetic patients leads to a decrease in the content of heparin sulfate in GBM, which in turn leads to changes in the permeability of negatively charged macromolecules (such as albumin), resulting in proteinuria (6, 8). In addition, the increased levels of reactive oxygen species induced by hyperglycemia not only degrades GAGs on glycocalyxes but also activates matrix metalloproteinases to induce proteolysis of sugar yeast, thereby leading to glycocalyx shedding and promotes kidney disease (9). Therefore, maintaining or restoring the integrity of the glycocalyx appears to be a promising therapeutic target.

In addition to being an anticoagulant, heparin also has unique properties of high negative charge and high heterogeneity, can interact with various proteins, exert a variety of nonanticoagulant activities, and show new and unexpected therapeutic effects in DN (10). Firstly, low molecular weight heparin (LMWH) can improve blood rheology and renal microcirculation in diabetic patients, delaying glomerular sclerosis, and reducing intrarenal circulation resistance (11). Secondly, heparin has certain antiinflammatory and antioxidant functions, which can inhibit inflammatory responses and renal cell damage (12, 13, 14). Furthermore, heparin or heparin analogs can act as heparanase inhibitors *in vivo*, reducing the degradation of HS on GBM, and protecting the glomerular barrier (8, 15). Moreover, LMWH can bind to the receptor for advanced glycation end product (RAGE) as an antagonist of RAGE and improve the various indicators of DN (16). Nevertheless, the cellular and molecular mechanisms involved (functional glycan chain structure and target protein) are not fully understood and need to be further explored and elucidated.

HS participates in various pathophysiological processes by interacting with a variety of proteins. Therefore, it is necessary to understand how exogenous heparin/heparin analogs affect changes in the proteome for revealing abovementioned mechanism. In this study, we analyzed the renal proteome of mice in the DN and LMWH treatment groups and found that LMWH could significantly promote the high expression of fatty acid–binding protein 1 (FABP1), Acaa1b, Acox2, Hmgcs2, and Pltp downstream of the peroxisome proliferator–activated receptors (PPARs) pathway, indicating that LMWH could promote the PPAR pathway to protect the kidney. LMWH could protect the HS of renal tubular cells from heparanase destruction and maintain HS-mediated endocytosis of FABP1 in renal tubules and the activation level of PPAR. Furthermore, we also characterize the sequence characteristics and molecular mechanisms of HS and FABP1 interactions.

## Results

### Polymerization distribution analysis of LMWH

LMWH is a U.S. Food and Drug Administration-approved drug that contains a mixture of oligosaccharides. We prepared the LMWH from unfractionated as described under “Experimental procedures.” The prepared LMWH was analyzed by size-exclusion chromatography (SEC), and its degree of polymerization was mainly distributed in dp4-dp24 (Fig. S1). The molecular weight distribution of the LMWH prepared by us is consistent with the literature report (17).

### LMWH can alleviate renal pathological changes in DN mice

**Figure 1:**
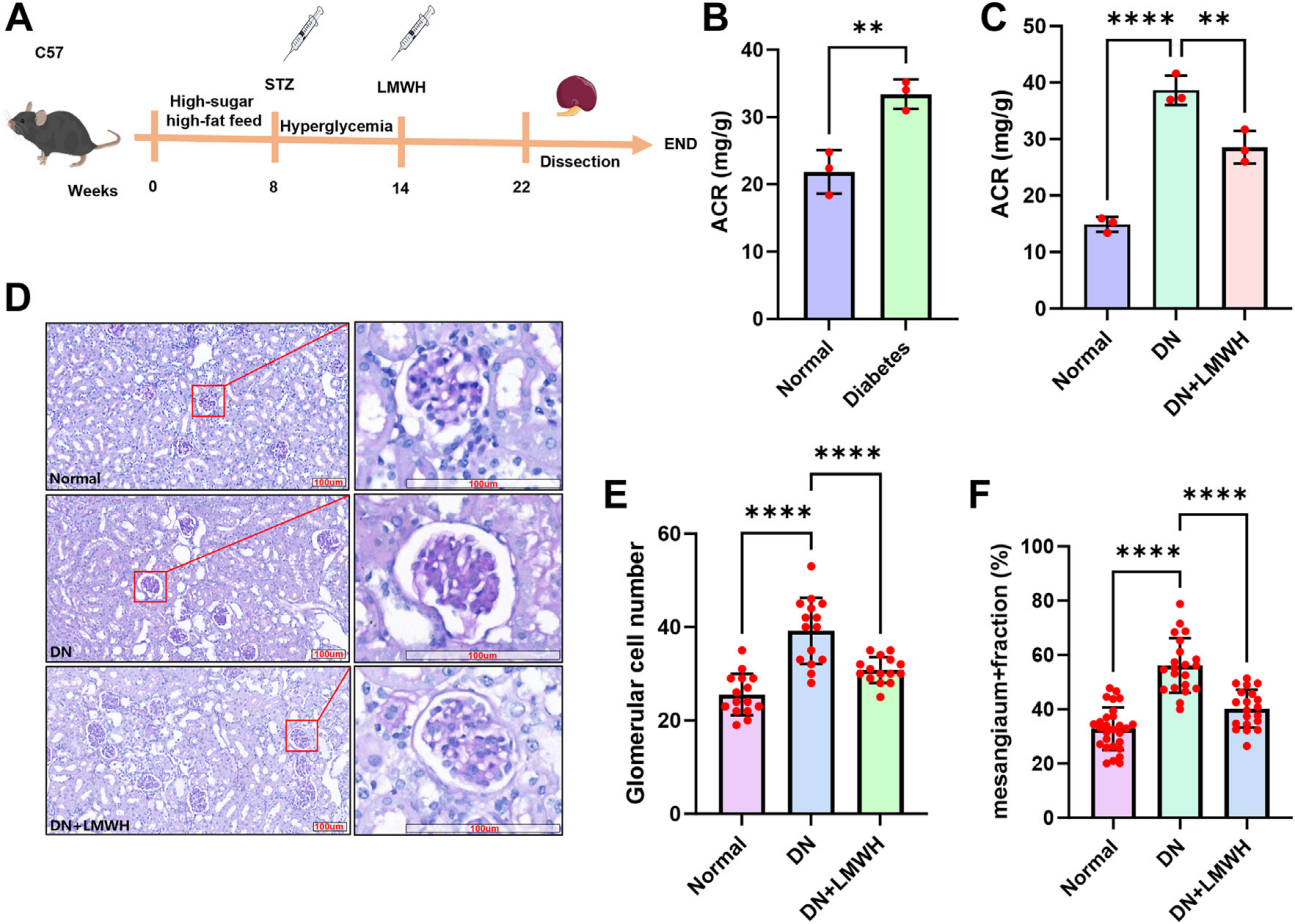
**Evaluation of the therapeutic effects of LMWH in DN.***A*, schematic diagram of of the establishment of the DN mouse model and drug administration. *B*, ACR values in mice after 6 weeks of diabetes (n = 3). *C*, ACR values in mice after 8 weeks of heparin treatment (n = 3). *D*, PAS staining of thin kidney sections (n = 3). *E*, glomerular cell number (n = 3, five glomeruli were randomly selected from each section for counting). *F*, mesangium fraction (n = 3, five glomeruli were randomly selected from each section to calculate the ratio of positive staining area to glomerular area). The data from the two cohorts were subjected to analysis using the independent samples *t* test. For comparisons involving multiple groups, one-way ANOVA followed by Dunnett's post hoc test was employed. Significance levels are denoted as follows: ∗ *p* < 0.05, ∗∗ *p* < 0.01, ∗∗∗ *p* < 0.001, and ∗∗∗∗ *p* < 0.0001. ACR, albumin/creatinine ratio; DN, diabetic nephropathy; LMWH, low molecular weight heparin; PAS, periodic acid shiff.

The establishment of the animal DN model and the experimental process of LMWH intervention are shown in Figure 1*A*. After 8 weeks of high-fat, high-sugar diet, streptozocin (75 mg/kg) was injected for three consecutive days to construct T2DM. The fasting blood glucose was greater than 16 mmol/L 1 week later, suggesting that the diabetes model was successful. After 6 weeks of continued feeding, urine albumin/creatinine ratio (ACR) was significantly higher than that of the normal group, indicating the development of DN (Fig. 1*B*). We found that LMWH administration for 8 weeks significantly decreased ACR of the mice with DN (Fig. 1*C*). Kidney sections were stained with periodic acid shiff (PAS) (Fig. 1*D* and five glomeruli per piece were randomly selected per piece to assess glomerular hypertrophy by cell counting (Fig. 1*E*). The relative number of pixels (pink or red area) divided by the total glomerular area was measured to assess mesangial interstitial dilation (Fig. 1*F*). The evaluation results showed that LMWH treatment significantly delayed the progression of DN, which is basically consistent with the results of previous studies (16).

### Proteomic analysis

**Figure 2:**
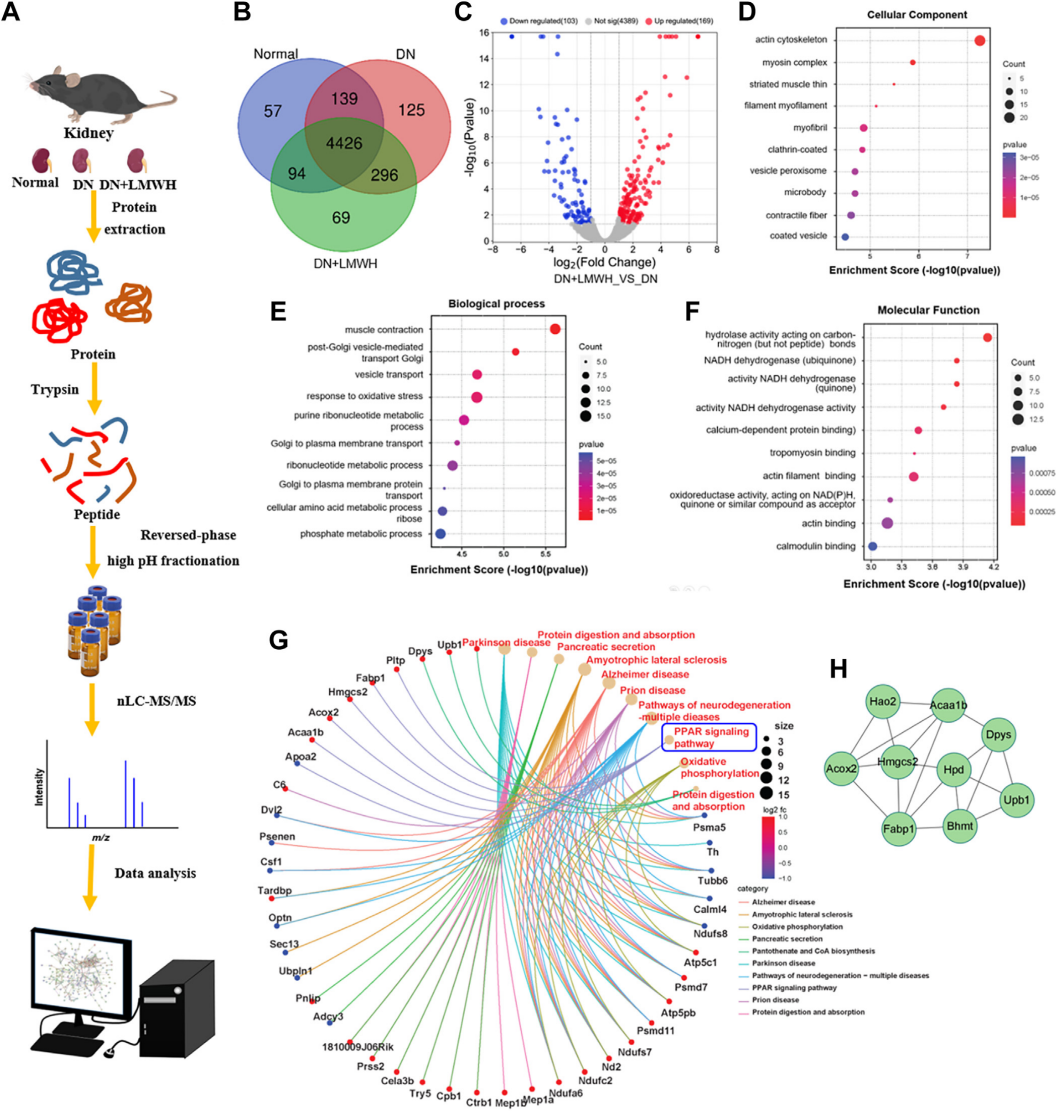
**Renal proteomic analysis and functional enrichment analysis of differentially expressed proteins.***A*, schematic diagram of renal proteomics. *B*, number of proteins identified by LC-MS/MS label-free proteomics analysis (n = 3). *C*, *volcano plot* of quantitative proteomics results in the DN group and LMWH treatment group (n = 3). *D*, GO biological process. *E*, GO molecular function. *F*, GO cellular component. *G*, KEGG analysis. *H*, Subnetworks of PPI. DN, diabetic nephropathy; GO, gene ontology; LMWH, low molecular weight heparin; KEGG, Kyoto encyclopedia of genes and genomes; PPI, protein–protein interaction.

The protective effects of heparin or heparin analogs on the diabetic kidney have been well studied (8, 16, 18, 19). However, its mechanism has not fully understood, especially, the effects of LMWH intervention on the renal proteome have not been reported. It is well known that proteins play an irreplaceable role in physiology/pathology, and *in vivo* HS or exogenous LMWH may function by interacting with various target proteins. To further understand the mechanism of LMWH in improving DN, we analyzed it by renal proteomics (Fig. 2*A*). A total of 4955 proteins were identified using label-free quantification techniques and quantitatively compared with at least two replicates (Fig. 2*B*). In the quantitative comparison between the LMWH treatment group and the DN model group, the expression levels of 272 proteins were significantly changed, with fold changes greater than two or less than 0.5, *p* < 0.05 (Table S1). Among them, 169 proteins were upregulated and 103 proteins were downregulated in the LWMH treatment group (Fig. 2*C*). It is unclear how many of these altered proteins are closely related to the role of heparin, so it makes sense to look for key proteins and explore the causes of their expression changes and the mechanism of their effects.

### Bioinformatics analysis and screening of key proteins

To screen the key proteins involved in kidney protection among the changed proteins, we performed GO and Kyoto encyclopedia of genes and genomes (KEGG) analysis and visualization of the 272 proteins that produced the changes in https://www.bioinformatics.com.cn, an online platform for data analysis and visualization.

GO analysis can define and describe the function of genes and proteins, and the GO database is divided into three categories: cellular component, biological process (BP), and molecular function. The enriched cellular component mainly includes actin cytoskeleton, myocoagulin complex, myofibrils, *etc.* (Fig. 2*D*). Actin cytoskeleton provides structural and functional support, generates a framework for cells and their connection with the extracellular matrix, and the complex regulation of podocytes actin cytoskeleton is the basis for maintaining an intact glomerular filtration barrier (20). The main BP involved were the response to oxidative stress, Golgi apparatus-related transport, purine metabolism, amino acid metabolism, *etc.* (Fig. 2*E*). Among them, oxidative stress plays a very important role in the course of DN, which directly leads to renal interstitium, glomeruli, and renal podocytes damage, and then damages the function of the kidney (21). The enriched molecular function mainly contains NADH dehydrogenase activity and protein binding (Fig. 2*F*). Among them, NADH dehydrogenase is the main component of mitochondrial respiratory chain complex I. Studies have shown that hyperglycemia can lead to mitochondrial dysfunction, which in turn inhibits respiratory chain complex I, which leads to the production of large amounts of reactive oxygen species to induce oxidative stress (22). GO analysis showed that LMWH may protect diabetic kidneys by participating in maintaining the renal actin backbone, protecting mitochondrial function, regulating oxidative stress, and other functions.

The KEGG analysis results are shown in Figure 2*G*. Among them, the enrichment of pathways is related to neurodegenerative diseases such as Parkinson's and Alzheimer's disease, which is not difficult to understand, because high blood glucose and high blood lipids can lead to mitochondrial dysfunction, which in turn is closely related to neurodegenerative diseases (23). We note that the lipid sensor–PPAR pathway, which regulates whole-body energy metabolism and is involved in diabetes and DN, was enriched. PPAR can reduce hyperglycemia-induced oxidative stress and apoptosis, and improve endothelial and podocyte function (24). In addition, PPAR agonists (such as fibrates for PPARα and glitazone for PPARγ) have been used for decades to treat dyslipidemia and diabetes (25). The PPAR signaling pathway and the location of the differentially expressed proteins Fabp1, Acaa1b, Acox2, Hmgcs2, Pltp, and Apoa2 involved in the pathway are shown in Fig. S2. These six proteins are the downstream target proteins of PPAR, and all them except Apoa2 were upregulated in the LMWH treatment group, suggesting that LMWH would protect diabetic kidneys by enhancing the PPAR pathway.

To further screen for key proteins affected by LMWH, the protein interaction network (medium confidence (0.4)) of 272 proteins was analyzed on STRING, the protein–protein interaction was visualized using Cytoscape software (https://cytoscape.org/) (Fig. S3), and subnet enrichment was performed using the MCODE plugin. The participating PPAR pathways Acox2, Hmgcs2, Fabp1, and Acaa1b were clustered into the same subnetwork (Fig. 2*H*), and all five proteins belonged to the downstream target genes of PPARα (26). Among them, intracellular FABP1 concentration was positively correlated with the transactivation of PPARα and PPARγ (27, 28, 29). In addition, FABP1 is thought to be a renal endogenous antioxidant that can inhibit tubulointerstitial damage (30). FABP1 is mainly expressed in the proximal renal tubule of the kidney, and the FABP1 filtered out by the glomeruli in the internal circulation is reabsorbed in the renal tubule, so the increase of FABP1 in the kidney is not necessarily due to the increase in expression, but may also be caused by the endocytosis of the filtered FABP1 by the proximal tubule, so treatment with LMWH may cause endocytosis of FABP1 that affects the renal tubules. Therefore, the protein FABP1 in the PPAR pathway was selected as a key protein in the influence of LMWH to conduct subsequent mechanism studies.

### Verification of FABP1, Acox2, Hmgcs2, PLTP, and Acaa1b expression changes

**Figure 3:**
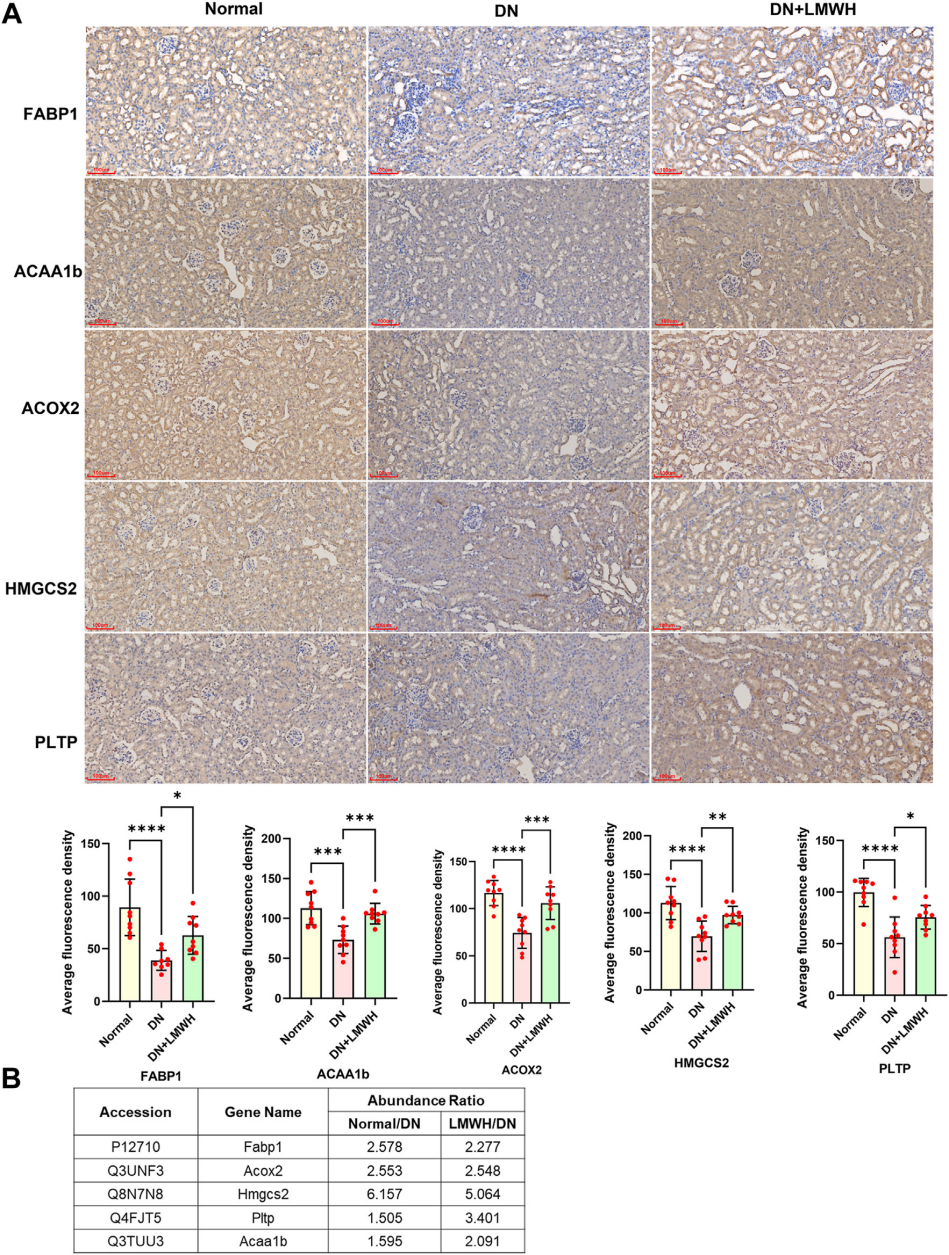
**Protein expression changes of FABP1, Acox2, Hmgcs2, and Acaa1b, PLTP.***A*, immunohistochemical staining of FABP1, Acox2, Hmgcs2, and Acaa1b, PLTP and quantification of average fluorescence intensity (n = 3, 20× magnification, three fields of view were selected for each kidney section for quantification). *B*, information on changes in abundance of five proteins in label-free proteome quantitation. For comparisons involving multiple groups, one-way ANOVA followed by Dunnett's post hoc test was employed. Significance levels are denoted as follows: ∗*p* < 0.05, ∗∗*p* < 0.01, ∗∗∗*p* < 0.001, and ∗∗∗∗*p* < 0.0001. FAB1, fatty acid–binding protein 1.

To further confirm the impact of LMWH treatment on the protein expression levels of FABP1, Acox2, Hmgcs2, Fabp1, and Acaa1b, we employed immunohistochemistry (IHC) to assess the actual expression levels of FABP1, Acox2, Hmgcs2, PLTP, and Acaa1b in the kidneys of mice. The results of IHC analysis were calculated with Image-Pro Plus 6.0 to calculate the average absorbance of positive staining of sections, and three fields of view were randomly taken for quantification per section (Fig. 3*A*). Compared with the DN group, the expressions of FABP1, Acox2, Hmgcs2, PLTP, and Acaa1 in the LMWH treatment group were significantly upregulated, which was close to that in the normal group. The results of IHC analysis were consistent with the results of proteome quantification (Fig. 3*B*), indicating that LMWH treatment could reverse the expression levels of FABP1, Acox2, Hmgcs2, PLTP, and Acaa1b in DN to some extent.

### Changes in the HS chain on glycocalyx of renal tubular cells affect their endocytosis to FABP1

Since FABP1 is expressed in the proximal tubule of the kidney and FABP1 involved in the internal circulation is also reabsorbed in the renal tubule, the reduced loss of FABP1 protein in the internal circulation is speculated to be one of the mechanisms by which LMWH upregulated its levels. We used human renal cortical proximal convoluted tubular epithelial cells (HK-2) treated with high-glucose-high-fat to establish a cell model.

**Figure 4:**
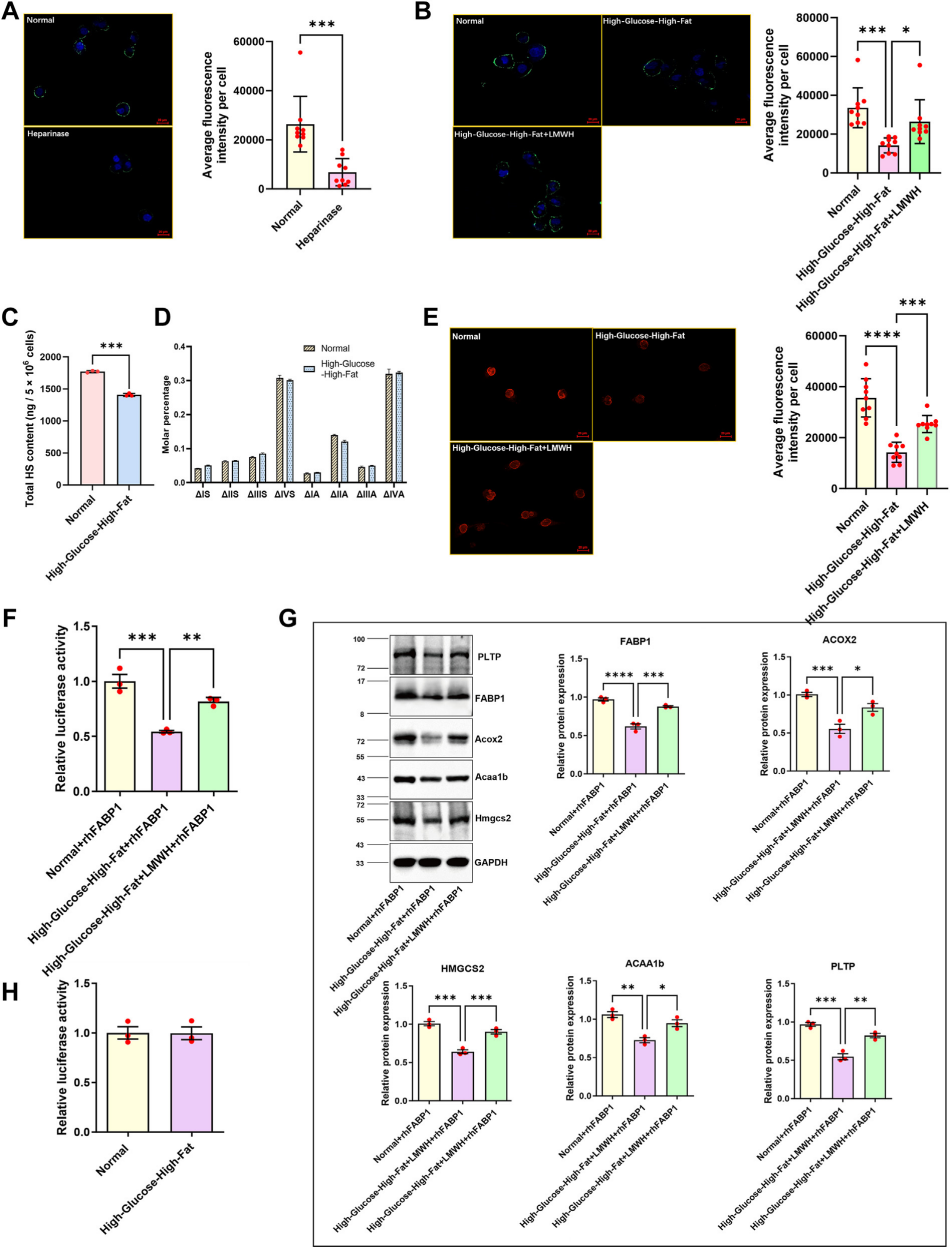
**Visualization and fluorescence quantification of HK-2 cell surface HS and intracellular FABP1.***A*, superpositively charged green fluorescent protein (ScGFP) labels highly negatively charged components on the cell surface. The *green color* diminished after incubation with heparinase (*bottom*), suggesting that HS is the dominant negatively charged species in HK-2. The scale bar represents 20 μm. *B*, visualization and fluorescence quantification of HS on the cell surface after high-glucose-high-fat treatment and high-glucose-high-fat + LMWH treatment with HK-2. The scale bar represents 20 μm. *C*, results of quantification of HS on the surface of HK-2 cells by LC-MRM (n = 3). *D*, composition of HS disaccharides on the surface of HK-2 cells (n = 3, ΔIS: ΔGlcA2S-GlcNS6S, ΔIIS: ΔGlcA-GlcNS6S, ΔIIIS: ΔGlcA2S-GlcNS, ΔIVS: ΔGlcA-GlcNS, ΔIA: ΔGlcA2S-GlcNAc6S, ΔIIA: ΔGlcA-GlcNAc6S, ΔIIA: ΔGlcA2S-GlcNAc6S, ΔIIIA: ΔGlcA2S-GlcNAc, ΔIVA: ΔGlcA-GlcNAc). *E*, visualization and fluorescence quantification of HK-2 endocytosis FABP1. The change of red fluorescence revealed that LMWH could reverse the endocytosis of FABP1 by HK-2 in a high-glucose-high-fat environment to a certain extent (n = 3, three fields of view were selected and the fluorescence of all cells in each field of view was quantified). The scale bar represents 20 μm. *F*, assess the effect of altered intracellular FABP1 levels on PPAR signaling using luciferase reporter assays. *G*, Western blot analysis of target gene proteins downstream of PPAR. *H*, analysis of the effect of high-glucose-high-fat on PPAR activation. The data from the two cohorts were subjected to analysis using the independent samples *t* test. For comparisons involving multiple groups, one-way ANOVA followed by Dunnett's post hoc test was employed. Significance levels are denoted as follows: ∗*p* < 0.05, ∗∗*p* < 0.01, ∗∗∗*p* < 0.001, and ∗∗∗∗*p* < 0.0001. FAB1, fatty acid–binding protein 1; HS, heparan sulfate; LC, liquid chromatography; LMWH, low molecular weight heparin; MRM, multiple reaction monitoring; PPAR, peroxisome proliferator–activated receptor.

Superpositively charged GFP (scGFP) was used to observe the highly negatively charged fraction on the surface of HK-2 cells. After heparinase treatment, the green fluorescence on the surface of HK-2 cells was significantly attenuated, indicating that the highly negatively charged components on the surface of HK-2 cells were HS (Fig. 4*A*). The results of quantification of fluorescence intensity showed that the high negatively charged glycan linkages on the surface of HK-2 cells were significantly reduced by the high-glucose-high-fat treatment，and the LMWH treatment group effectively protected the glycan chains on the cell surface from degradation (Fig. 4*B*). HS consists of eight different repeating disaccharide units with different sulfated and *N*-acetylated substitutions, so quantification of eight disaccharides can directly reflect the amount of cellular HS. To further reveal the structural changes in HS at the molecular level, eight disaccharides were quantitatively analyzed using LC/MS-multiple reaction monitoring (MRM) with stable isotope internal standards. The results showed that the levels of eight disaccharides in high-glucose-high-fat group was significantly lower than those in the normal group (Fig. 4*C*), indicating that the high-glucose-high-fat treatment would destroy the HS chain on the cell surface. However, its sulfation modification level is not affected, as the molar percentage of each disaccharide does not change significantly (Fig. 4*D*). In addition, in the endocytosis experiment, HK-2 in the normal group recruited a considerable amount of recombinant human FABP1 (rhFABP1) through endocytosis, and the rhFABP1 in HK-2 endocytosis in the high-glucose-high-fat group was significantly reduced, while this phenomenon was reversed to a certain extent in the LMWH treatment group (Fig. 4*E*), indicating that LMWH protects HS from disruption and plays an important role in mediating HK-2 endocytosis FABP1.

### Changes in endocytic FABP1 affect the level of activation of PPAR

Endogenous FABP1 is known to be a coactivator of PPAR, which can promote the transport of PPAR ligands such as free fatty acids to PPARs, thereby enhancing transcriptional regulation. To verify the effect of changes in endocytotic FABP1 on cellular PPAR activation, we utilized the PPAR-Luc luciferase reporter containing multiple PPAR binding sites to detect the activation level of PPAR in HK-2 cells. First, the PPAR-Luc luciferase reporter plasmid was transfected into HK-2 cells, and then treated with rhFABP1 with different administrations, and the luciferase activity was detected, and the results are shown in Figure 4*F*. The results showed that luciferase activity decreased and PPAR activation level decreased after high-glucose-high-fat treatment, and luciferase activity and PPAR activation level were reversed after LMWH combined treatment. The results showed that endocytosed FABP1 could affect the activation level of PPAR. At the same time, we also performed Western blot analysis on five PPAR downstream target gene proteins that were upregulated in the proteome (Fig. 4*G*), and the results showed that the activation of PPAR upregulated the target gene proteins, which was consistent with the results of animal experiments.

It is known that the high glucose environment increases the protein O-GlcNAcylation, and the abnormal O-GlcNAcylation of the protein plays a crucial role in the etiology and progression of diabetes and diabetic complications (31). The O-GlcNAcylation of PPAR may alter its transcriptional activity (32), to further evaluate the direct effect of high-glucose-high-fat treatment on PPAR, we did not significantly change the activation level of PPAR in the detection of luciferase activity in high-glucose-high-fat treated HK-2 cells, indicating that the high-glucose-high-fat environment did not directly affect the activation of PPAR (Fig. 4*H*).

### Protective effect of FABP1 on HK-2 cells in a high-glucose-high-fat environment

**Figure 5:**
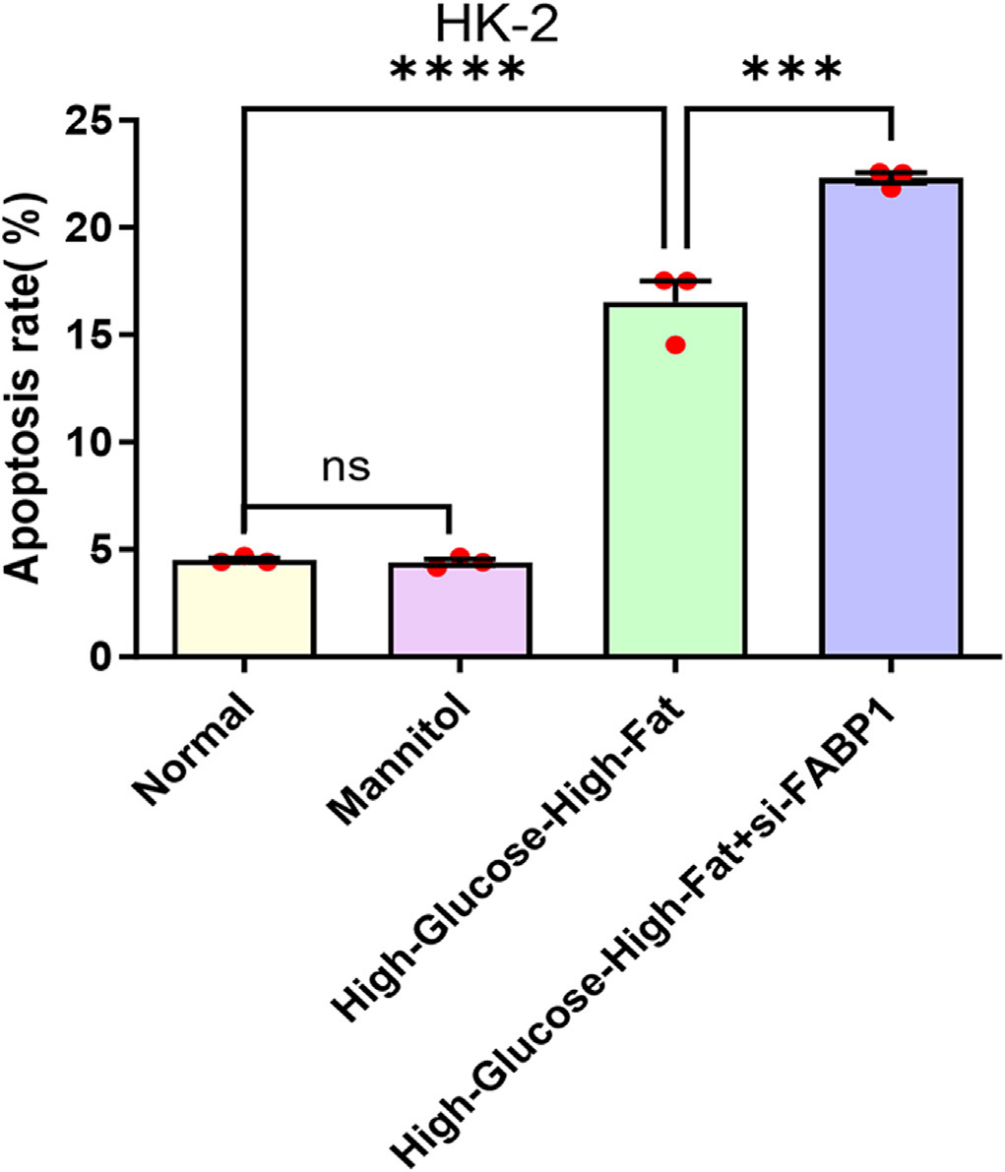
**Effect of silencing FABP1 on apoptosis of HK-2 cells in a high-glucose-high-fat environment.** For comparisons involving multiple groups, one-way ANOVA followed by Dunnett's post hoc test was employed. Significance levels are denoted as follows: ∗ *p* < 0.05, ∗∗ *p* < 0.01, ∗∗∗ *p* < 0.001, and ∗∗∗∗*p* < 0.0001. FAB1, fatty acid–binding protein 1.

To verify the role of FABP1 in DN, we used a cell model to silence FABP1 and detect changes in apoptosis of HK-2 cells in a high-glucose-high-fat environment, as shown in Figure 5. There was no significant change in the apoptosis rate in the mannitol treatment group with the same osmotic pressure as the high glucose medium, indicating that the osmotic pressure had no effect on the apoptosis of HK-2. The apoptosis of HK-2 cells increased significantly after high-glucose-high-fat stimulation, and the apoptosis rate of HK-2 cells in the high-glucose-high-fat environment after FABP1 silencing further increased, indicating that FABP1 had a protective effect on HK-2 cells in the high-glucose-high-fat environment.

### Effects of high-glucose-high-fat on HS synthesis and degrading enzymes in HK-2

**Figure 6:**
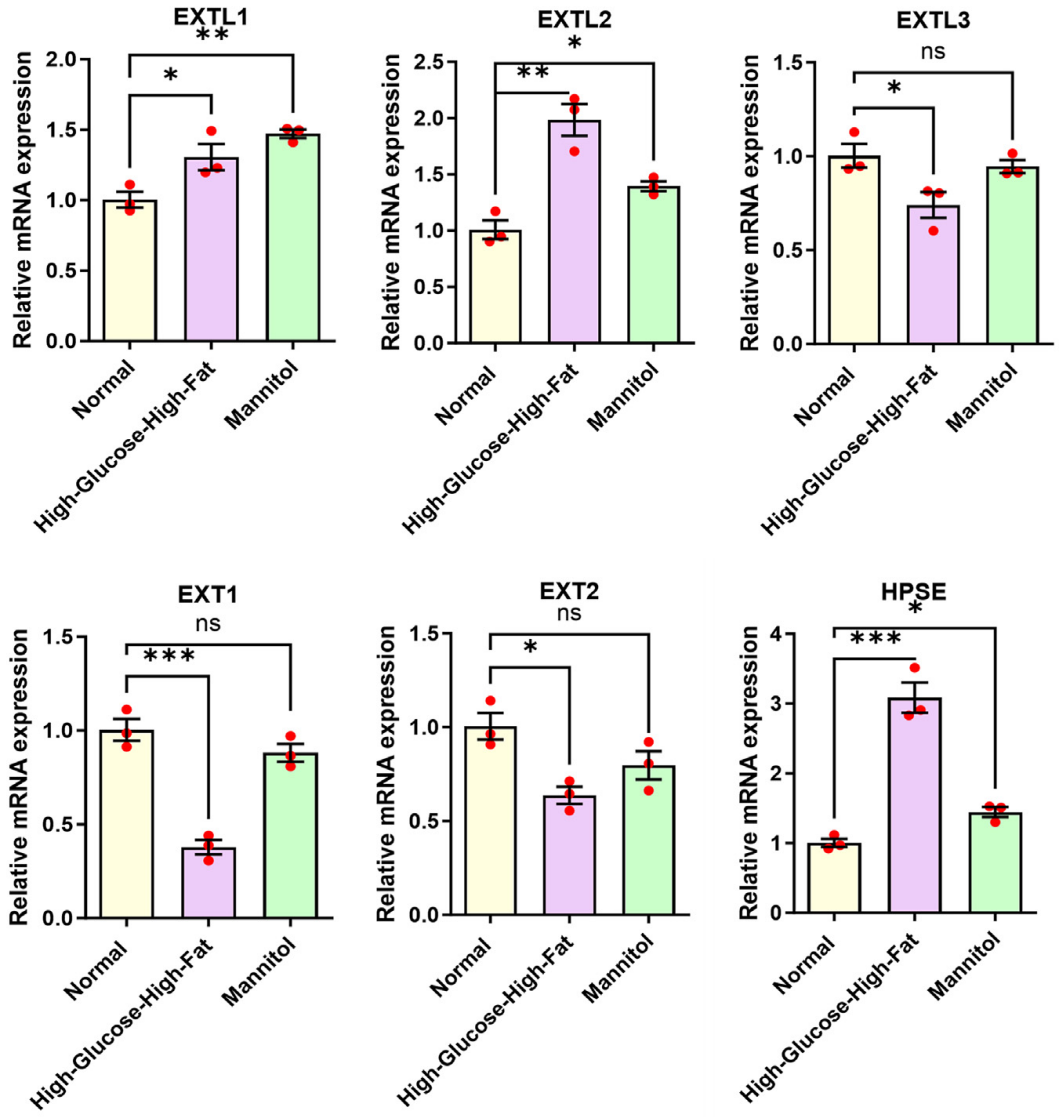
**Effects of high-glucose-high-fat on HS synthase and degrading enzyme in HK-2 cells.** For comparisons involving multiple groups, one-way ANOVA followed by Dunnett's *post hoc* test was employed. Significance levels are denoted as follows: ∗ *p* < 0.05, ∗∗ *p* < 0.01, ∗∗∗ *p* < 0.001, and ∗∗∗∗ *p* < 0.0001. HS, heparan sulfate.

We investigated the expression changes of HK-2 cells involved in HS synthase and HS degrading enzymes using quantitative RT-PCR to further understand the possible mechanism of HS shedding on the surface of HK-2 cells exposed to high-glucose-high-fat (Fig. 6). The findings indicated a significant upregulation in the expression of glycosyltransferases exostosin-like 1 and exostosin-like 2 in HK-2 cells subjected to high-glucose-high-lipid conditions. Conversely, the expression levels of exostosin-1 and exostosin-2 were downregulated, implying that high-glucose-high-fat conditions influence HS biosynthesis in cells. However, the current data did not exhibit a clear trend, making it difficult to ascertain whether the synthesis is ultimately promoted or inhibited. Nevertheless, the HS-degrading enzyme heparanase (HSPE) exhibited elevated expression, elucidating the primary cause of reduced HS levels on the surface of HK-2 cells within a hyperglycemic and hyperlipidemic milieu. Moreover, we assessed the alterations in the expression of HS synthase and degradative enzymes under the influence of mannitol (which mimics the osmotic pressure of high glucose) as illustrated in Figure 6, to explore the potential impact of osmotic pressure on HS depletion. It was observed that HSPE expression was markedly upregulated, although the effect of osmotic pressure on HSPE expression was comparatively less pronounced than that in the high-glucose-high-fat environment.

### Molecular mechanism of binding between FABP1 and heparin

**Figure 7:**
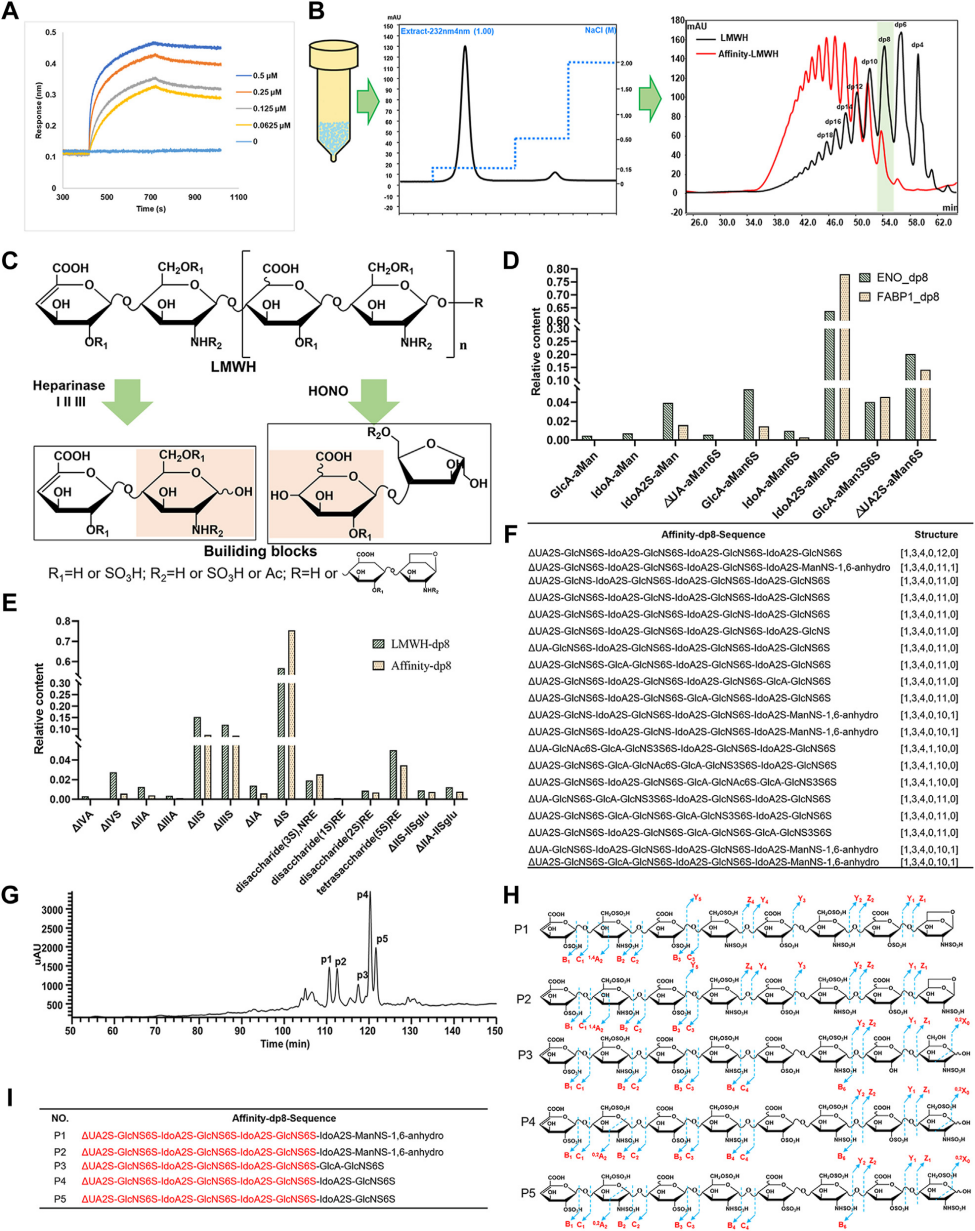
**Validation of FABP1–HS interactions and characterization of oligosaccharide structures involved in the interactions.***A*, BLI. *B*, FABP1 affinity chromatography separated affinity LMWH and SEC analysis affinity LMWH polymerization degree changes. *C*, schematic of complete enzymatic hydrolysis and HONO degradation of LMWH. *D*, comparison of the relative content of disaccharides after HONO degradation of LMWH-dp8 and affinity-dp8. *E*, comparison of the relative content of disaccharides after complete enzymatic hydrolysis of LMWH-dp8 and affinity-dp8. *F*, TOP20 sequence obtained by Sep-GAG software. *G*, SAX chromatogram of affinity-dp8. *H*, MS/MS sequencing results of the five major components of affinity-dp8. *I*, Sep-GAG combined with MS/MS sequencing to obtain sequences of the five components of affinity-dp8. BLI, biolayer interferometry; FAB1, fatty acid–binding protein 1; GAG, glycosaminoglycan; HONO, nitrous acid; HS, heparan sulfate; LMWH, low molecular weight heparin; MS/MS, tandem mass spectrometry; SAX, strong anion exchange chromatography; SEC, size-exclusion chromatography.

With advances in analytical methods, the specific veil of GAG–protein interactions is being lifted. To elucidate the sequence characteristics of HS binding to FABP1, we used our previously developed Sep-GAG software combined with offline strong anion exchange chromatography (SAX)-tandem mass spectrometry (MS/MS) methods for characterization (33). Firstly, our biolayer interferometry (BLI) results showed that the affinity between FABP1 and LMWH was very strong (K_D_ = 1.53 × 10^-9^ mol/L), indicating that FABP1 belonged to HSBP (Fig. 7*A*).

As shown in Figure 7*B*, LMWH was first fractionated by FABP1 affinity chromatography, with low salt solutions salts eluting nonaffinity components and high salt solutions eluting as affinity components. SEC was used to compare the changes in the polymerization degree of affinity components, nonaffinity components and LMWH, where the polymerization degree of affinity component oligosaccharide chains favored octasaccharide (dp8) and larger oligosaccharide chains, indicating that FABP1 has a certain length dependence on the binding of oligosaccharide chains. Therefore, we used affinity chromatography to enrich dp8 for population sequencing, in which complete enzymatic hydrolysis and nitrite degradation can provide information on the disaccharide composition units information of affinity dp8. Unfortunately, because enzymatic hydrolysis can well retain sulfate modification information and the hexosamine information well, it destroys the epimeric information of the C-5 uronic acid by forming unsaturated double bonds at positions 4,5 of the uronic acid, and nitrite degradation can compensate for this deficiency (Fig. 7*C*). The disaccharide information after complete enzymatic hydrolysis is shown in Figure 7*D*, and the ΔIS (ΔUA2S-GlcNS6S) content in FABP1-affinity dp8 exceeds the ΔIS content in LMWH-dp8 by 18.8%. The results of nitrite degradation showed that the content of IdoA2S-aMan6S in ABP1-affinity dp8 exceeds the content of IdoA2S-aMan6S in LMWH-dp8 by 14.2% (Fig. 7*E*). The information of the disaccharide constituent units indicated that FABP1 was more inclined to the IdoA2S-GlcNS6S structure of the junction and LMWH. The top 20 theoretical affinity dp8 sequences revealed by Seq-GAG software were present in Figure 7*F*. To obtain accurate oligosaccharide sequences, we first used SAX to further isolate affinity-dp8, which was characterized by MS/MS. A total of five major chromatographic peaks (P1-P5) were isolated from SAX (Fig. 7*G*), and the oligosaccharide sequences characterized by MS/MS are shown in Figure 4*H*, and the MS/MS mass spectra were shown in Fig. S4. Combined with the Sep-GAG sequencing and MS/MS results, the sequence structure of five affinity-dp8 (Fig. 7*I*) was obtained, and they had a common sequence "ΔUA2S-GlcNS6S-IdoA2S-GlcNS6S," where ΔUA2S could be IdoA2S or GlcA2S in the parent glycan chain. Therefore, we conclude that HS of tubular cells should contain a structure of "IdoA2S/GlcA2S-GlcNS6S-IdoA2S-GlcNS6S-IdoA2S-GlcNS6S" by which mediating FABP1 endocytosis.

**Figure 8:**
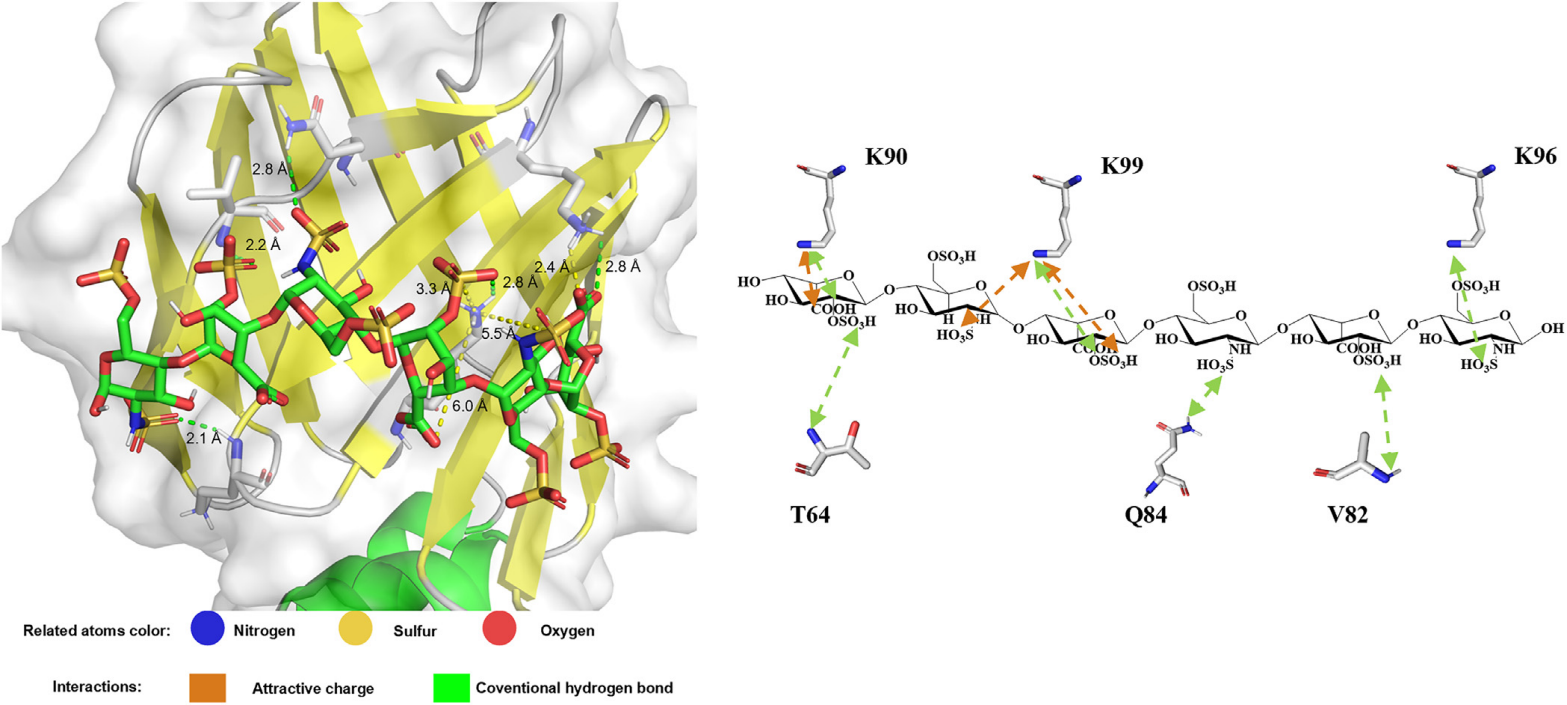
**Molecular docking of dp6 and FABP1.***Left panel* shows the complex of the FABP1 (shown in part) and the hexasaccharide, visualized using AutoDock simulation. The *right panel* demonstrates the contributions of the protein and oligosaccharide binding motifs and the types of interactions (*orange dashed arrows* represent electrostatic attractions, and *green dashed arrows* represent hydrogen bonds). FAB1, fatty acid–binding protein 1.

Furthermore, we employed GlycoTorch Vina to model the interaction between heparin hexasaccharide (PDB: 1FQ9) and FABP1 (PDB: 7FYA) (Fig. 8). The calculated optimal binding free energy was −3.6 kcal/mol, facilitated through both electrostatic and hydrogen bonding interactions. Key lysine residues (K90, K96, and K99) in FABP1 were essential for ligand binding, establishing critical electrostatic contacts. Each monosaccharide unit within the hexasaccharide chain contributed to the binding, with *N*-sulfation and 2-*O*-sulfation playing crucial roles.

## Discussion

DN is an important cause of end-stage renal disease, affecting 40% of patients with diabetes. DN may occur in patients with previous hyperglycemic exposure, even if glycemic control is reasonable (16, 34). The protective effect of heparin analogs on the diabetic kidney has long been reported clinically, which can restore the barrier function of the GBM by protecting glomerular capillary HS, and can also be used as an antagonist of RAGE to treat DN (8, 16, 35). Therefore, further exploration of the mechanism of action of heparin analogs is very valuable for the development of drugs to treat the diabetic kidney. HS can mediate many physiological processes by interacting with a variety of HSBPs (proteases, growth factors, cytokines, chemokines, and adhesion molecules) (5). LMWH can protect against the degradation of cellular glycocalyx HS in long-term hyperglycemic environments, thereby affecting HSBPs.

PPARs are ligand-activated nuclear transcription factors that include three subtypes: PPARα, PPARβ, and PPARγ. It plays an important role in BPs such as lipid metabolism, glucose homeostasis, cell cycle progression, cell differentiation, inflammation, and extracellular matrix remodeling (36). Many studies have shown that the availability of selective agonists and antagonists of PPARs may provide new avenues for the treatment of DN. Among them, the PPAR-α agonist fenofibrate prevented DN by improving the function of db/db mouse endothelial cells and inhibiting M1 macrophages (37). PPARγ agonists such as troglitazone (Rezulin), pioglitazone (ACTOS), and rogradone (Avandia) reduce insulin resistance, hyperinsulinemia, and hyperglycemia in diabetics (36). Therefore, studying the PPAR pathway may provide a new way to regulate DN.

In this study, we verified in animal experiments that LMWH treatment can improve DN and revealed for the first time the changes in the renal proteome after LMWH treatment. The downstream proteins FABP1, Acaa1b, Acox2, Hmgcs2, and PLTP of the PPAR pathway were upregulated, so LMWH may improve DN by enhancing the PPAR pathway. FABP1 is mainly expressed in the proximal renal tubules, filtered through the glomeruli in circulation, and then reabsorbed in the renal tubules. Therefore, the elevated level of FABP1 in urine can be used as a marker of DN (38). Importantly, FABP1 has a protective effect in acute kidney injury and chronic kidney disease and may reduce glomerular injury in the early stages of IgAN (39, 40). Therefore, in this study, FABP1 was investigated as a representative HSBP. LMWH has been proved to protect the glomerular endothelial HS and the normal barrier function of the glomerulus, thereby reducing protein loss (8). In this study, we visualized the HS of tubular epithelial cells by fluorescent protein labeling and quantified the changes in HS content by the stable isotope internal standard liquid chromatography-MRM. Immunofluorescence localization analysis was used to elucidate the mechanism by which HS is involved in the endocytic recruitment of FABP1 in renal tubules, and LMWH could protect glycocalyx HS and reduce the degradation induced by high glucose and high lipids. Furthermore, studies have shown that FABP1 is a coactivator of PPAR-mediated gene expression, as FABP1 acts as a cytoplasmic channel for PPARα and PPARγ agonists such as fatty acids (27, 41), and in this study, we utilized a luciferase reporter gene to monitor the activation level of PPAR, demonstrating that changes in endocytic FABP1 levels affect the activation level of PPAR. Therefore, LMWH indirectly promotes the PPAR pathway by protecting cellular HS and increasing the content of intracellular FABP1 levels to delay the progression of DN. Probably, due to the complexity of the HS structure and the lack of advanced analytical tools, few detailed mechanisms of HSBP and HS interaction have been elucidated. We enriched the oligosaccharide chains of FABP1 interaction by affinity chromatography and used Seq-GAG software combined with off-line SAX-MS/MS to characterize the HS structures participating in the interaction, including IdoA2S/GlcA2S-GlcNS6S-IdoA2S-GlcNS6S sequences. In addition, we also predicted the protein sites involved in the interaction by molecular docking technology, in which K90, K96, and K99 made major contributions to the binding of the two. This study revealed a novel mechanism by which LMWH can delay the progression of DN by promoting the PPAR pathway and elucidated the molecular mechanism of FABP1 interaction with HS, providing new insights into understanding the role of heparin in the pathogenesis of DN and the development of appropriate treatments.

## Experimental procedures

### Preparation of LMWH

The preparation of LMWH by β-elimination follows the protocol we previously reported (17). Briefly, we first prepared heparin benzathonium salt; the obtained heparin benzathonium salt was then redissolved in dichloromethane, and heparin benzyl ester was prepared by adding benzyl chloride (40 °C, 12 h); next, 1 g of heparin benzyl ester was incubated with 25 ml of 0.1 M sodium hydroxide at 55 °C for 2 h for depolymerization. Finally, LMWH was obtained by methanol precipitation and dialysis, and its polymerization distribution was analyzed by SEC. SEC analysis was performed on a Thermo Scientific Vanquish UHPLC System, and the ACQUITY UPLC Protein BSH SEC Columns (300 mm × 4.6 mm and 150 mm × 4.6 mm tandem, Waters) were used to separate the samples. Mobile phase was composed of 50 mM ammonium formate in 20% methanol. The flow rate was set at 75 μl/min for a total analysis time of 75 min.

### DN mouse models and grouping

Male mice (C57BL/6J, age 4 weeks, body weight 12–14 g, purchased from Beijing SPF Biotechnology Co., Ltd) were housed in an environmentally controlled room with 35 to 55% humidity at 25 °C ± 0.5 °C, 12 h: 12 h light/dark cycle and acclimated to this environment 1 week before the experiment. They were then randomized into a T2DM model group (fed a high-fat diet, 10% sucrose, 10% lard, 5% cholesterol) and a normal control group (fed normal chow). After 8 weeks, the model group was intraperitoneally injected with 75 mg/kg streptozotocin (Sigma) for three consecutive days, and the fasting blood glucose >16.0 mmol/L after 1 week was the standard for molding. After the model group continued the high-fat diet for 4 weeks, the measured ACR was significantly different from that of the normal group, and renal lesions were judged. The DN model group was randomly divided into DN group and LMWH treatment group (subcutaneous injection of HP 100 IU/pcs/day). After 8 weeks, ACR was measured and then euthanized for further analysis. The kidney was harvested for proteomics and sectioning (PAS staining). All experiments were approved by the Institutional Animal Care and Use Committee of the Scientific Investigation Committee of Shandong University.

### Histological evaluation

Mice are perfused with 20 ml normal saline immediately after euthanasia, the kidneys were immersed in 4% paraformaldehyde for fixation; paraffin is embedded and cut into 4-μm thick sections. Staining was performed with PAS. The method of analyzing and evaluating glomerular hypertrophy and mesangial expansion with Image software refers to the published literature (42), and in briefly, three stained glomeruli (three mice per group) per slice are randomly selected to analyze the degree of glomerular hypertrophy. Semiquantitative assessment of mesangial stromal dilation is assessed by measuring the relative number of pixels (pink or red areas) divided by the total glomerular area.

### Renal protein extraction, tryptic digestion, and peptide prefractionation

Kidney protein extraction, tryptic digestion, and peptide prefractionation steps were performed according to the reported literature method (43). Briefly, the isolated tissue was ground and dissolved in T-PER Tissue Protein Extraction Regent (Thermo Fisher Scientific) containing a protease and phosphatase inhibitor (Thermo Fisher Scientific), followed by ultrasonic fragmentation for 60 s (3-s on and 3-s off, amplitude 25%). Samples were centrifuged at 12,000*g* to remove residual tissue. The protein concentration was measured by bicinchoninic acid method. An equal amount of protein was denatured with denaturation buffer (8 M urea, 0.1 M Tris–HCl, pH 8.5), then the sample was reduced with 10 mM DTT for 4 h at 37 °C, followed by an additional 30 min of 50 mM iodoacetamide alkylation in the dark at room temperature (all on a 30 kDa ultrafiltration membrane to facilitate buffer replacement). Then added trypsin (*w*/*w* = 1:50) to each filter tube, incubated at 37 °C for 12 hand ultrafiltrated to yield peptides. Finally, the polypeptide was preseparated by high pH reversed-phase, vacuum concentrated and lyophilized, and stored at −80 °C for later use.

### Nano LC-MS/MS analysis for label-free proteomics

Peptides were analysed using a nano system (Thermo Fisher Scientific, EASY-nLC1200) coupled to a Nano Orbitrap Fusion Lumos Tribrid mass spectrometer system (Thermo Fisher Scientific). Briefly, the prefractionated peptides were dissolved in 0.1% formic acid (FA) in 2% acetonitrile, first collected on a nanoViper C18 silica gel column Acclaim PepMap RSLC (75 μm × 2 cm, 3 μm, 100 A, Thermo Fisher Scientific), and after washing, the peptide was transferred to a nanoViper C18 silica gel column Acclaim PepMap RSLC (75 μm × 25 cm, 2 μm, Thermo Fisher Scientific) for separation. Mobile phases A (0.1% FA) and B (80% acetonitrile, 0.1% FA) with flow rates of 300 nl/min. Gradient procedure: 0 to 4 min, 2% to 10% B; 4 to 44 min, 10% to 28% B; 44 to 55 min, 28% to 38% B; 55 to 60 min, 38% to 55% B; 60 to 65 min, 55% to 95% B; 65 to 75 min, and 95% B. Finally, the eluted peptide was sprayed into the mass spectrometer by nanospray ion source, and the cycle-time data-dependent acquisition mode was adopted. The main scan interval is 3 s; scan range: 200 to 2000 *m/z*; 400 *m/z* resolution of 60,000; in the Orbitrap detector with a resolution of 15,000, the precursor ions intensity threshold greater than 4.0e5 in the quadrupole were selected for MS/MS fragmentation analysis with a normalized collision energy of 30%, and the dynamic exclusion time was 25 s to avoid repeated screening of polypeptides.

### Proteome data analysis

All RAW files were processed using Proteome Discoverer (https://www.thermofisher.cn/order/catalog/product/OPTON-31105) (v.2.3, Thermo Fisher Scientific) with the Sequest HT search engine, parameters: trypsin specificity, up to two missed lyses; digested peptide lengths from 4 to 144 Da; oxidation of methionine and acetyl at the N terminus for dynamic modification and carbamidomethyl of cysteines in static modification; after quantification, we define proteins with fold change > 2, *p* value <0.05 as differentially expressed proteins.

### Bioinformatic analysis

GO enrichment analysis of differentially expressed proteins and KEGG pathway enrichment analysis and visualization were performed in https://www.bioinformatics.com.cn, an online platform for data analysis and visualization, with adjusted *p* < 0.05 was set as the cut-off criteria. The protein–protein interaction network was constructed from the STRING database and visualized by Cytoscape software (V3.10.0).

### Immunohistochemistry

Paraffin-embedded kidney blocks were cut into 4-μm thick sections for IHC. Rabbit monoclonal FABP1 antibody (diluted 1:4000; Arkham) was used as the primary antibody and horseradish peroxidase anti-rabbit IgG (Boster) as the secondary antibody. After the secondary antibody incubation is completed, DAB working solution (Boster) is added drop by drop, counterstained with Meyer's haematoxylin (Boster) and observed under a microscope. The brown–yellow area is positive.

### Cell culture and treatment

HK-2 cells (human proximal tubular epithelial cells) were purchased from Boster Biological Technology co.ltd. HK-2 cells were cultured in Iscove's modified Dulbecco's medium supplemented with 10% fetal bovine serum at 37 °C with 5% CO_2_. HK-2 cells were cultured with 45 mM glucose and a gradient concentration (50, 100, 200, 300, and 400 μM) of palmitic acid for 48 h and cell viability was measured using the methyl thiazolyl tetrazolium kit.

### Visualization of glycocalyx on the surface of HK-2 cells

Cell surface negatively charged HS was labeled with the highly positively charged fluorescent protein scGFP for visualization (44). Cells cultured under normal and high-glucose-high-fat (45 mM glucose, 300 μM PA) for 48 h were incubated with a scGFP incubator for 5 min and then washed three times with PBS to remove free scGFP. After staining nuclei with 4’,6-diamidino-2-phenylindole (Solarbio), visualization was performed using super-resolution laser scanning confocal microscopy (LSM900) (Carl Zeiss) and the ZEISS LSM image browser.

### Analysis of HS component disaccharides

Analysis of HS component disaccharides was performed as previously described (45). Briefly, cells were rinsed three times with PBS, then tissue protein extract was added and sonicated in an acoustic bath for 10 min, then added a certain amount of stable isotopically labeled eight heparin disaccharides, added 400 μl of heparin complete digestion buffer and 20 mIU each of heparinase I, II, and III, incubated at 37 °C for 12 h, and continued incubation for 12 h after adding an equal amount of enzyme. The stable isotopically labeled disaccharides were synthesized chemoenzymatically as described previously (46). The resulting disaccharides are obtained by a 3 kDa ultrafiltration membrane, freeze-dried, and labeled with 2-aminoacridone reductive amination. LC-MS–MRM was performed using an ExionLC UPLC system connected to a SCIEX Triple Quad 5500+ mass spectrometer. A Kinetex C18 column (2.6 μm, 150 × 2.1 mm, Agela Technologies) was used at a column temperature of 45 °C. MRM transitions for 2-aminoacridone –labeled disaccharides refer to reported protocols (47).

### Real-time quantitative PCR

Total RNA from HK-2 cells was extracted by Trizol method. Determine the purity and concentration of RNA utilizing an ultramicrovolume spectrophotometer. Reverse transcription of RNA to complementary DNA using HiScript reverse transcriptase for HPSE, exostosin-like 1, exostosin-like 2, exostosin-like 3, exostosin-1, exostosin-2 are shown in Table S1. The data were analyzed using the 2^-ΔΔCt^ analysis method.

### Visualization of FABP1 endocytosis in living cells

HK-2 cells are seeded in a 35 mm dish with a 14 mm glass-bottom well and randomly divided into two groups after 24 h for further incubation for 48 h in 45 mM glucose, 300 μM PA and normal medium, respectively. The cells were then incubated with histidine (His)-tagged human recombinantly expressed FABP1 (10 μg/ml) for 1 h and washed 3 times with PBS to remove excess FABP1. The cells were then fixed with 4% paraformaldehyde for 20 min, washed with PBS, and permeabilized with 0.2% Triton X-100 for 15 min at room temperature. After blocking with 5% bovine serum albumin, the primary antibody (anti-His tag) and the secondary antibody were added sequentially, both were incubated for 1 h at room temperature, and washed four times with PBST after 1 h. Finally, staining with 4’,6-diamidino-2-phenylindole was performed. Visualisation was performed using super-resolution laser scanning confocal microscopy (LSM900, Carl Zeiss AG) and the ZEISS LSM image browse.

### Detection of PPAR activation levels

The activation level of PPAR was quantified by the assay of PPAR luciferase reporter plasmid. HK-2 cells were seeded into 24-well culture plates, 7.5∗10^4^ cells/well and incubated at 37 °C for 24 h. PPAR luciferase reporter plasmid was transfected according to the protocol of Polyplus Transfection Reagent. After transfection, the cells were divided into normal group, high-glucose and high-fat group, and high-glucose and high-fat combined with LMWH treatment group, respectively, after 48 h, the cells were incubated with His-labeled rhFABP1 (10 μg/ml) for 24 h, and then the dual luciferase was detected according to the protocol of the TransDetect double-luciferase reporter assay kit.

### Western blotting

Add 3 ml of prechilled PBS to the cell culture flask, wash the cells with gentle shaking for 1 min, and remove the wash, repeat three times. Add 400 μl of T-PER protein extraction reagent containing 1% protease inhibitor and 0.5% phosphatase inhibitor and scrape the cells with a cell scraper to extract the total protein. Centrifuge the cell solution at 12,000*g* at 4 °C for 10 min, collect the supernatant and determine the total protein concentration using the bicinchoninic acid protein assay kit. The sample was mixed with loading buffer and boiled for 5 min. Electrophoresis parameters were 12.5% SDS-PAGE gel, 20 μg sample load, and 200 v constant pressure electrophoresis, protein transfer to polyvinylidene fluoride membrane parameters, 200 mA constant current for 90 min. After blocking with 5% skim dry milk, the primary antibody and secondary antibody incubation were performed sequentially. The VILBER Fusion FX Imaging System was used for imaging, and Image J (https://imagej.net/ij/index.html) was used for data processing.

### Apoptosis assay

HK-2 cells were seeded into 6-well plates and cultured overnight, 3.5∗10^5^ cells/well. Si-FABP1 (sense, GGGAAGCACUUCAAGUUCATT; antisense, UGAACUUGAAGUGCUUCCCTT) was transfected with jetPRIME buffer and then treated with high glucose and high fat for 48 h. Cells were collected and assayed using flow cytometry.

### Biolayer interferometry

BLI is a well-established method for validating protein–GAG interactions. LMWH is first biotinylated by the method we have reported (48). Biotinylated LMWH was coupled to a streptavidin sensor. FABP1 was diluted with PBS buffer to form a gradient of five concentrations (0, 0.0625 μM, 0.125 μM, 0.25 μM, 0.5 μM). ForteBio Octet Red96e (ForteBio) was used to detect LMWH and FABP1 interactions according to our published protocols (49).

### FABP1-agarose affinity chromatography enrichment for affinity heparin oligosaccharides

The preparation of the protein affinity column and the enrichment method for affinity oligosaccharides followed our previously reported protocol, with appropriate optimizations implemented. (33). Briefly, the heparin-blocked FABP1 was conjugated to the activated cyanogen bromide activated Sepharose 4B (4 °C overnight) to prepare affinity columns. LMWH dissolved in loading buffer (10 mM Tris–HCl) was loaded onto affinity columns, nonaffinity oligosaccharides were eluted with low salt buffer (0.15 M NaCl, 1 mM Tris–HCl, pH = 6.5), and affinity oligosaccharides were eluted with high salt buffer (2 M NaCl, 1 mM Tris–HCl, pH = 6.5). Finally, the collected affinity oligosaccharides were separated and collected by polymerization using the Waters 1.7 μm SEC 125 Å Column (4.6 mm × 150 mm and 4.6 mm × 300 mm tandem).

### Acquisition of the dp8 theoretical sequences

The method used by the Seq-GAG software to infer theoretical sequences in mixed oligosaccharide chains uses the protocol we have reported (33). Briefly, the dp8 was subjected to enzymatic digestion and nitrous acid (HONO) degradation, respectively, to analyze its basic building blocks. dp8 was dissolved in 8.75 μl heparin digestion buffer (prepared by mixing sodium acetate/calcium acetate buffer), 5 mIU each of heparinase I, II, and III were added, incubated at 37 °C for 12 h, an equal amount of enzyme was added and incubation was continued for 12 h. Enzymatic digestion samples were analyzed using hydrophilic interaction chromatography MS/MS. HONO degradation of dp8 at pH 1.5 followed the protocol previously reported (50). HONO-degraded samples were analyzed by porous graphitized carbon chromatography-MS. The liquid analysis described above was performed on a Thermo Fisher Scientific Ultimate 3000UHPLC coupled to an LTQ-Orbitrap XL mass spectrometer. Finally, Seq-GAG，a software we developed， was used for sequence prediction.

### Off-line SAX and ESI-MS/MS

dp8 was further separated using SAX (Thermo Fisher Scientific, ProPac PA1 SAX column, 4 × 250 mm) and the components were collected by chromatographic peak. Mobile phase A was 0.2 M NaCl (pH 7.0), and mobile phase B was 2 M NaCl (pH 7.0). The gradient change of the mobile phases was 0% B for 5 min, 0% B to 30% B over 50 min, 30% B to 70% B over 100 min, 70% B to 100% B over 0.1 min, and 100% B for 10 min, and the total analysis time was 160 min. The collected components were desalted and analyzed using the Thermo LTQ-Orbitrap XL mass spectrometer for MS and MS/MS using the protocol we previously reported (51). The sample solvent and mobile phase consist of a 50% methanol solution (3 mM NaOH). The MS/MS parameters were set as follows: Iso width (m/z), 3.0; normalized collision energy, 50.0.

### Molecular docking

The docking between heparin hexasaccharide and FABP1 is performed using GlycoTorch Vina software (https://www.glycotorch.com/). The structure of heparin hexasaccharide is derived from the Protein Data Bank code 1FQ9, and FABP1 is derived from the NMR structure in the database (PDB: 7FYA). Before docking, hydrogen atoms were added to the protein structure and the Gasteiger charge was used. The docking range is the whole protein unrestricted docking, and the receptor adopts full flexible docking.

## Data availability

Proteome data are available *via* ProteomeXchange with identifier PXD045022. All relevant data generated during this study or analyzed in this manuscript (and its Supplement files) are available from the corresponding author on reasonable request.

## Supporting information

This article contains supporting information.

## Conflict of interest

The authors declare that they have no conflicts of interest with the contents of this article.

## References

[1] Alicic R.Z., Rooney M.T., Tuttle K.R. (2017). Diabetic kidney disease. Clin. J. Am. Soc. Nephrol..

[2] Afkarian M., Sachs M.C., Kestenbaum B., Hirsch I.B., Tuttle K.R., Hinnmelfarb J. (2013). Kidney disease and increased mortality risk in type 2 diabetes. J. Am. Soc. Nephrol..

[3] A/L B Vasanth Rao V.R., Tan S.H., Candasamy M., Bhattamisra S.K. (2019). Diabetic nephropathy: an update on pathogenesis and drug development. Diabetology Metab. Syndr..

[4] Pessentheiner A.R., Ducasa G.M., Gordts P. (2020). Proteoglycans in obesity-associated metabolic dysfunction and meta-inflammation. Front. Immunol..

[5] Shi D., Sheng A., Chi L. (2021). Glycosaminoglycan-protein interactions and their roles in human disease. Front. Mol. Biosci..

[6] Hiebert L.M. (2021). Heparan sulfate proteoglycans in diabetes. Semin. Thromb. Hemost..

[7] Maxhimer J.B., Somenek M., Rao G., Pesce C.E., Baldwin D., Gattuso P. (2005). Heparanase-1 gene expression and regulation by high glucose in renal epithelial cells: a potential role in the pathogenesis of proteinuria in diabetic patients. Diabetes.

[8] Lewis E.J., Xu X. (2008). Abnormal glomerular permeability characteristics in diabetic nephropathy: implications for the therapeutic use of low-molecular weight heparin. Diabetes Care.

[9] Korakas E., Ikonomidis I., Markakis K., Raptis A., Dimitriadis G., Lambadiari V. (2020). The endothelial glycocalyx as a key mediator of albumin handling and the development of diabetic nephropathy. Curr. Vasc. Pharmacol..

[10] Wang P., Chi L., Zhang Z., Zhao H., Zhang F., Linhardt R.J. (2022). Heparin: an old drug for new clinical applications. Carbohydr. Polym..

[11] Guo H., Gan L., Yu Z. (2001). The protective effect of low molecular weight heparin on early nephropathy in diabetic rats. Zhonghua Yi Xue Za Zhi.

[12] Abbadi A., Loftis J., Wang A., Yu M., Wang Y., Shakya S. (2020). Heparin inhibits proinflammatory and promotes anti-inflammatory macrophage polarization under hyperglycemic stress. J. Biol. Chem..

[13] Xu D., Young J., Song D., Esko J.D. (2011). Heparan sulfate is essential for high mobility group protein 1 (HMGB1) signaling by the receptor for advanced glycation end products (RAGE). J. Biol. Chem..

[14] Arnold K., Xu Y., Sparkenbaugh E.M., Li M., Han X., Zhang X. (2020). Design of anti-inflammatory heparan sulfate to protect against acetaminophen-induced acute liver failure. Sci. Transl. Med..

[15] Zhai S., Hu L., Zhong L., Tao Y., Wang Z. (2017). Low molecular weight heparin may benefit nephrotic remission in steroid-sensitive nephrotic syndrome via inhibiting elastase. Mol. Med. Rep..

[16] Myint K.M., Yamamoto Y., Doi T., Kato I., Harashima A., Yonekura H. (2006). RAGE control of diabetic nephropathy in a mouse model: effects of RAGE gene disruption and administration of low-molecular weight heparin. Diabetes.

[17] Guan Y., Xu X., Liu X., Sheng A., Jin L., Linhardt R.J. (2016). Comparison of low-molecular-weight heparins prepared from bovine lung heparin and porcine intestine heparin. J. Pharm. Sci..

[18] Jiao B., Zhang Y.H., Cheng Y.N., Gao J.J., Zhang Q.Z. (2011). A low-dose combination of valsartan and low molecular weight heparin better improved glomerular permeability than did high-dose monotherapy in rats with diabetic nephropathy. Drug Discoveries Ther..

[19] Cha J.J., Kang Y.S., Hyun Y.Y., Han S.Y., Jee Y.H., Han K.H. (2013). Sulodexide improves renal function through reduction of vascular endothelial growth factor in type 2 diabetic rats. Life Sci..

[20] Tian X., Ishibe S. (2016). Targeting the podocyte cytoskeleton: from pathogenesis to therapy in proteinuric kidney disease. Nephrol. Dial. Transplant..

[21] Sagoo M.K., Gnudi L. (2018). Diabetic nephropathy: is there a role for oxidative stress?. Free Radic. Biol. Med..

[22] Waldhart A.N., Muhire B., Johnson B., Pettinga D., Madaj Z.B., Wolfrum E. (2021). Excess dietary carbohydrate affects mitochondrial integrity as observed in brown adipose tissue. Cell Rep..

[23] Norat P., Soldozy S., Sokolowski J.D., Gorick C.M., Kumar J.S., Chae Y. (2020). Mitochondrial dysfunction in neurological disorders: exploring mitochondrial transplantation. Npj Regen. Med..

[24] Kim Y., Lim J.H., Kim M.Y., Kim E.N., Yoon H.E., Shin S.J. (2018). The adiponectin receptor agonist AdipoRon ameliorates diabetic nephropathy in a model of type 2 diabetes. J. Am. Soc. Nephrol..

[25] Montaigne D., Butruille L., Staels B. (2021). PPAR control of metabolism and cardiovascular functions. Nat. Rev. Cardiol..

[26] Rakhshandehroo M., Knoch B., Müller M., Kersten S. (2010). Peroxisome proliferator-activated receptor alpha target genes. PPAR Res..

[27] Wolfrum C., Borrmann C.M., Borchers T., Spener F. (2001). Fatty acids and hypolipidemic drugs regulate peroxisome proliferator-activated receptors alpha - and gamma-mediated gene expression via liver fatty acid binding protein: a signaling path to the nucleus. Proc. Natl. Acad. Sci. U. S. A..

[28] Wang G., Bonkovsky H.L., de Lemos A., Burczynski F.J. (2015). Recent insights into the biological functions of liver fatty acid binding protein 1. J. Lipid Res..

[29] Negishi K., Noiri E., Maeda R., Portilla D., Sugaya T., Fujita T. (2008). Renal L-type fatty acid-binding protein mediates the bezafibrate reduction of cisplatin-induced acute kidney injury. Kidney Int..

[30] Herman-Edelstein M., Scherzer P., Tobar A., Levi M., Gafter U. (2014). Altered renal lipid metabolism and renal lipid accumulation in human diabetic nephropathy. J. Lipid Res..

[31] Ma J.F., Hart G.W. (2013). Protein-GlcNAcylation in diabetes and diabetic complications. Expert Rev. Proteomics.

[32] Ji S., Park S.Y., Roth J., Kim H.S., Cho J.W. (2012). O-GlcNAc modification of PPARγ reduces its transcriptional activity. Biochem. Biophys. Res. Commun..

[33] Shi D., Sheng A., Bu C., An Z., Cui X., Sun X. (2022). A cluster sequencing strategy to determine the consensus affinity domains in heparin for its binding to specific proteins. Anal. Chem..

[34] Yamazaki T., Mimura I., Tanaka T., Nangaku M. (2021). Treatment of diabetic kidney disease: current and future. Diabetes Metab. J..

[35] Van der Pijl J.W., Lemkes K.H.P.J., Frolich M., Van der Woude F.J., Van der Meer F.J.M., Van Es L.A. (1999). Effect of danaparoid sodium on proteinuria, von Willebrand factor, and hard exudates in patients with diabetes mellitus type 2. J. Am. Soc. Nephrol..

[36] Guan Y., Breyer M.D. (2001). Peroxisome proliferator-activated receptors (PPARs): novel therapeutic targets in renal disease. Kidney Int..

[37] Feng X., Gao X., Wang S., Huang M., Sun Z., Dong H. (2021). PPAR-Alpha agonist fenofibrate prevented diabetic nephropathy by inhibiting M1 macrophages via improving endothelial cell function in db/db mice. Front. Med..

[38] Yamamoto T., Noiri E., Ono Y., Doi K., Negishi K., Kamijo A. (2007). Renal L-type fatty acid--binding protein in acute ischemic injury. J. Am. Soc. Nephrol..

[39] Wu J., Shao X., Shen J., Lin Q., Zhu X., Li S. (2022). Downregulation of PPARalpha mediates FABP1 expression, contributing to IgA nephropathy by stimulating ferroptosis in human mesangial cells. Int. J. Biol. Sci..

[40] Zuo N., Suzuki Y., Sugaya T., Osaki K., Kanaguchi Y., Wang L.N. (2011). Protective effects of tubular liver-type fatty acid-binding protein against glomerular damage in murine IgA nephropathy. Nephrol. Dial. Transplant..

[41] Furuhashi M., Hotamisligil G.S. (2008). Fatty acid-binding proteins: role in metabolic diseases and potential as drug targets. Nat. Rev. Drug Discov..

[42] Li S., Zheng L., Zhang J., Liu X., Wu Z. (2021). Inhibition of ferroptosis by up-regulating Nrf2 delayed the progression of diabetic nephropathy. Free Radic. Biol. Med..

[43] Jiang Y., Sun A., Zhao Y., Ying W., Sun H., Yang X. (2019). Proteomics identifies new therapeutic targets of early-stage hepatocellular carcinoma. Nature.

[44] Wang W.S., Han N.H., Li R.J., Han W.J., Zhang X.R., Li F.C. (2015). Supercharged fluorescent protein as a versatile probe for the detection of glycosaminoglycans in vitro and in vivo. Anal. Chem..

[45] Li G., Li L., Tian F., Zhang L., Xue C., Linhardt R.J. (2015). Glycosaminoglycanomics of cultured cells using a rapid and sensitive LC-MS/MS approach. ACS Chem. Biol..

[46] Wang Z.J., Arnold K., Xu Y.M., Pagadala V., Su G.W., Myatt H. (2020). Quantitative analysis of heparan sulfate using isotopically labeled calibrants. Commun. Biol..

[47] Wang Z.J., Arnold K., Dhurandhare V.M., Xu Y.M., Pagadala V., Labra E. (2022). Analysis of 3-O-sulfated heparan sulfate using isotopically labeled oligosaccharide calibrants (vol 94, pg 2950, 2022). Anal. Chem..

[48] Shi D., He P., Song Y., Cheng S., Linhardt R.J., Dordick J.S. (2022). Kinetic and structural aspects of glycosaminoglycan-monkeypox virus protein A29 interactions using surface plasmon resonance. Molecules.

[49] Chen Q., Li F., Wang H., Bu C., Shi F., Jin L. (2022). Evaluating the immunogenicity of heparin and heparin derivatives by measuring their binding to platelet factor 4 using biolayer interferometry. Front. Mol. Biosci..

[50] Gill V.L., Wang Q., Shi X., Zaia J. (2012). Mass spectrometric method for determining the uronic acid epimerization in heparan sulfate disaccharides generated using nitrous acid. Anal. Chem..

[51] Wang Z., Zhang T., Xie S., Liu X., Li H., Linhardt R.J. (2018). Sequencing the oligosaccharide pool in the low molecular weight heparin dalteparin with offline HPLC and ESI-MS/MS. Carbohydr. Polym..

