# Supplementary material for: Low molecular weight heparin promotes the PPAR pathway by protecting the glycocalyx of cells to delay the progression of diabetic nephropathy
Source: J Biol Chem. 2024 Jun 24;300(8):107493. doi: 10.1016/j.jbc.2024.107493 (PMC11301383; doi:10.1016/j.jbc.2024.107493)
Supplement: Supplementary data [file mmc1.docx]

# Low molecular weight heparin promotes the PPAR pathway by protecting the glycocalyx of cells to delay the progression of diabetic nephropathy

Bin Zhang, Changkai Bu, Qingchi Wang, Qingqing Chen, Deling Shi, Jian Liu, Qunye Zhang, and Lianli Chi

## Supplemental Material: Table of Contents

## Supplemental Figures

**Figure S1.** Size Exclusion Chromatography (SEC) analysis of LMWH

**Figure S2.** PPAR signaling pathway

**Figure S3.** Protein-protein interaction network (PPI) analysis of kidney perturbation proteins in DN mice during LMWH treatment

**Figure S4.** MS/MS spectra of affinity-dp8

**Table S1.** Information on differentially expressed proteins in label-free proteomics quantification

**Table S2**. Primer for the synthesis and degradation enzymes of HS


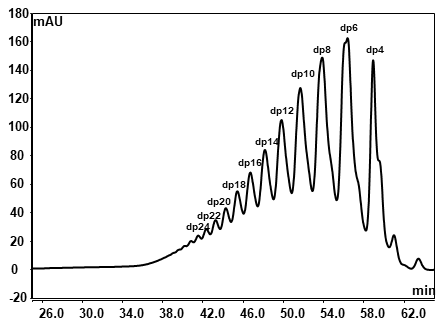


**Figure S1.** Size Exclusion Chromatography (SEC) analysis of LMWH

**
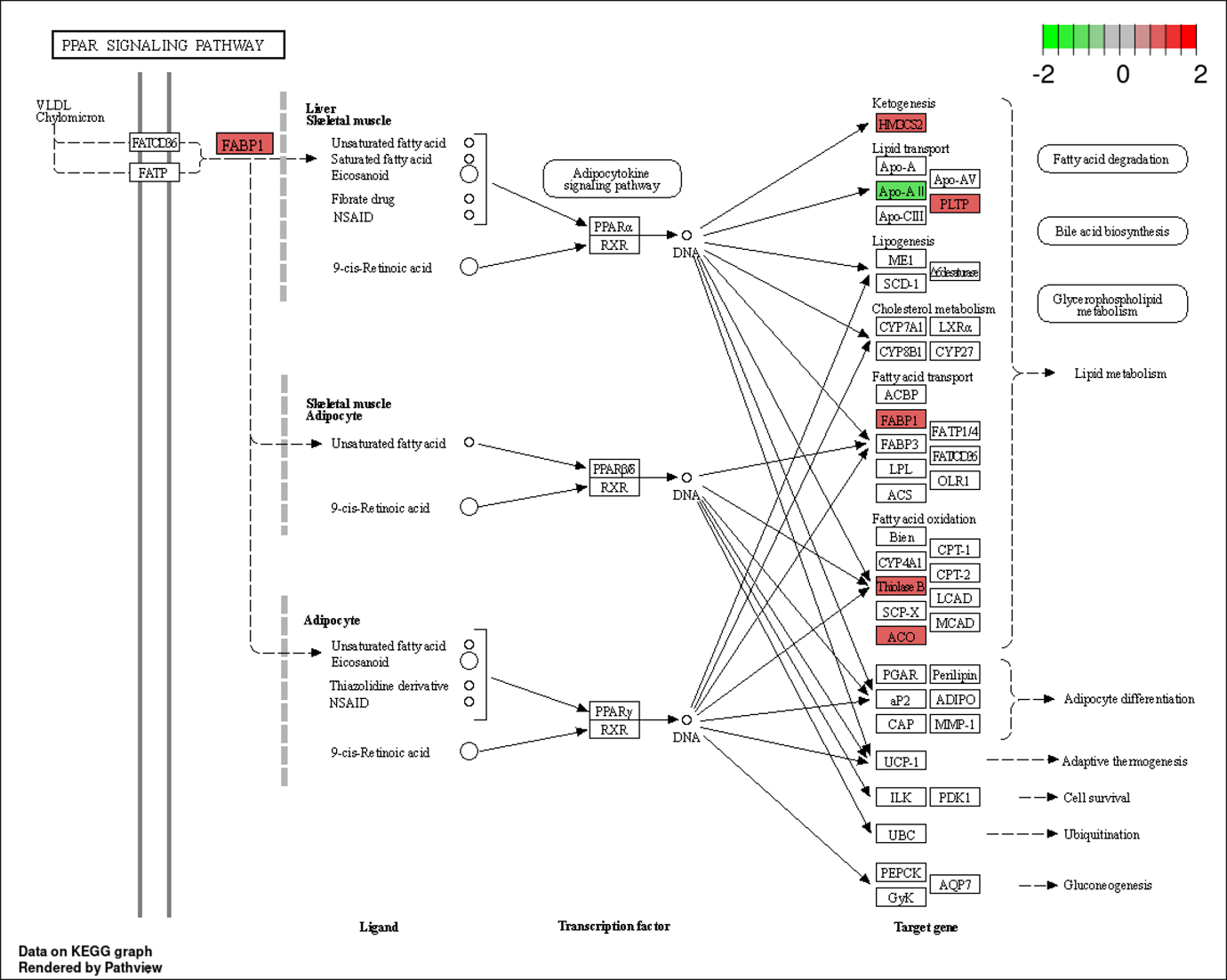
**

**Figure S2.** PPAR signaling pathway (03320). Red is the up-regulated protein after LMWH treatment, and green is the down-regulated protein. Signal pathway diagrams are available in https://www.bioinformatics.com.cn, an online platform for data analysis and visualization.


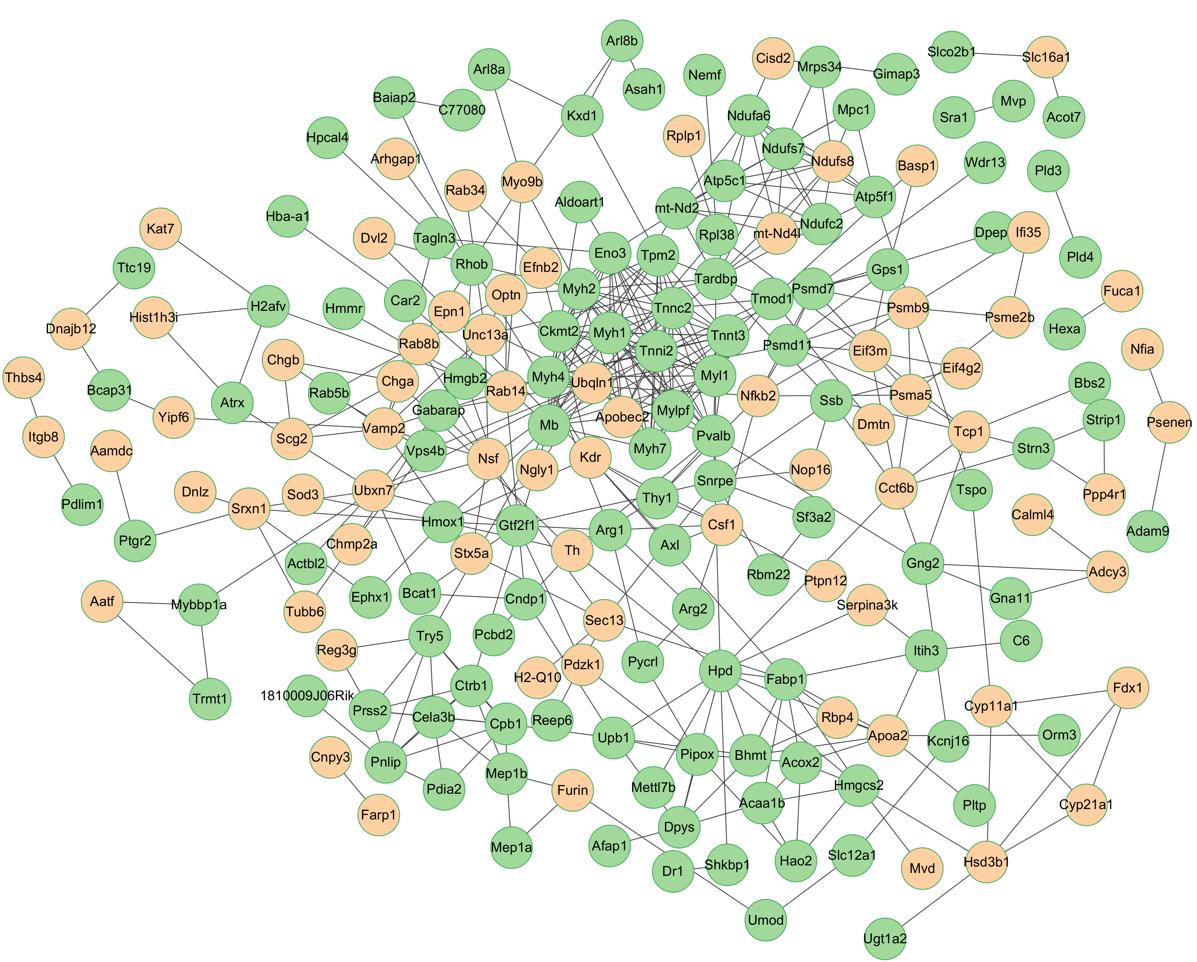


**Figure S3.** Protein-protein interaction network (PPI) analysis of kidney perturbation proteins in DN mice during LMWH treatment. Light green is upregulated protein and brownish yellow is downregulated protein. The PPI was analyzed by STRING and visualized by Cytoscape.


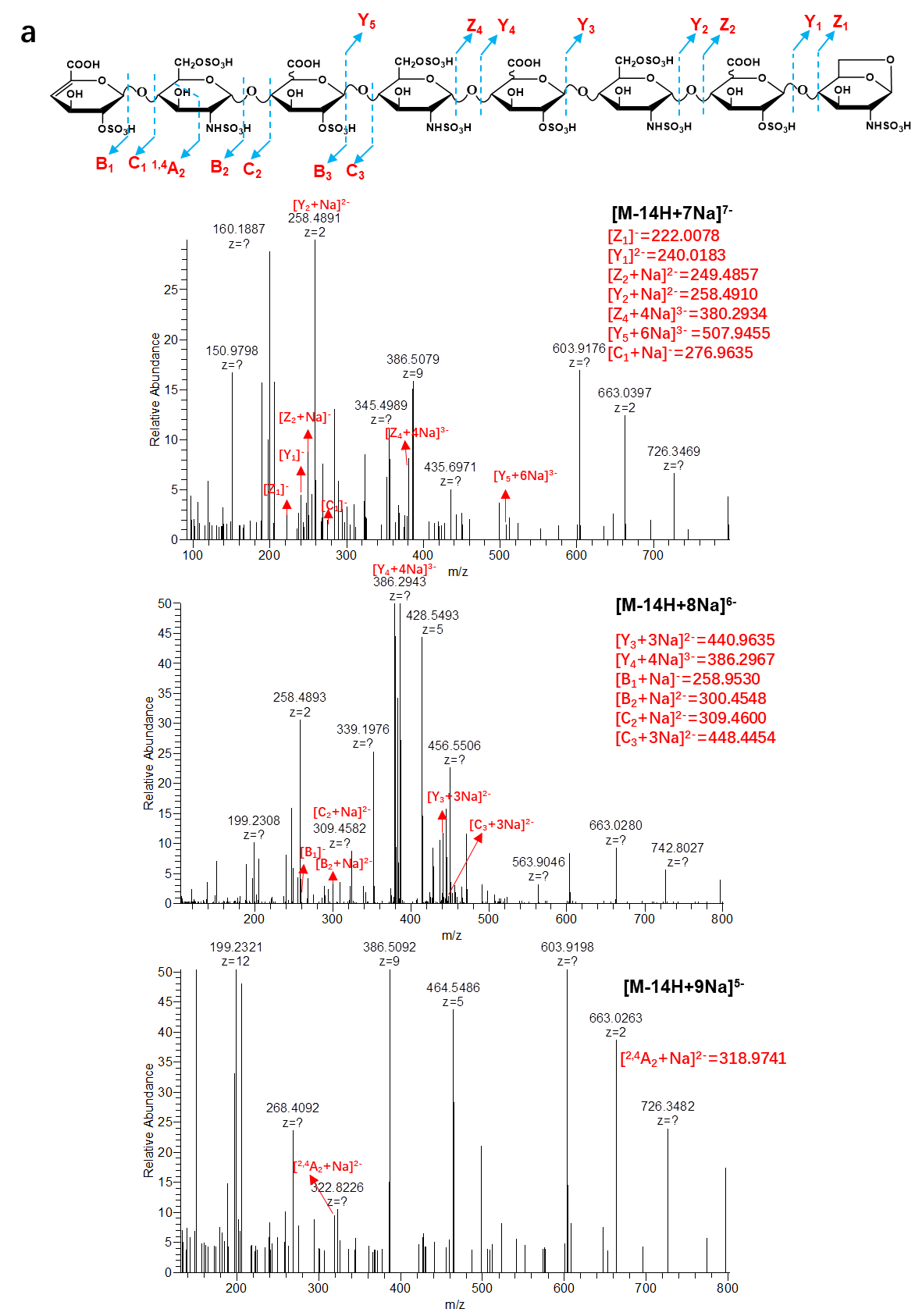


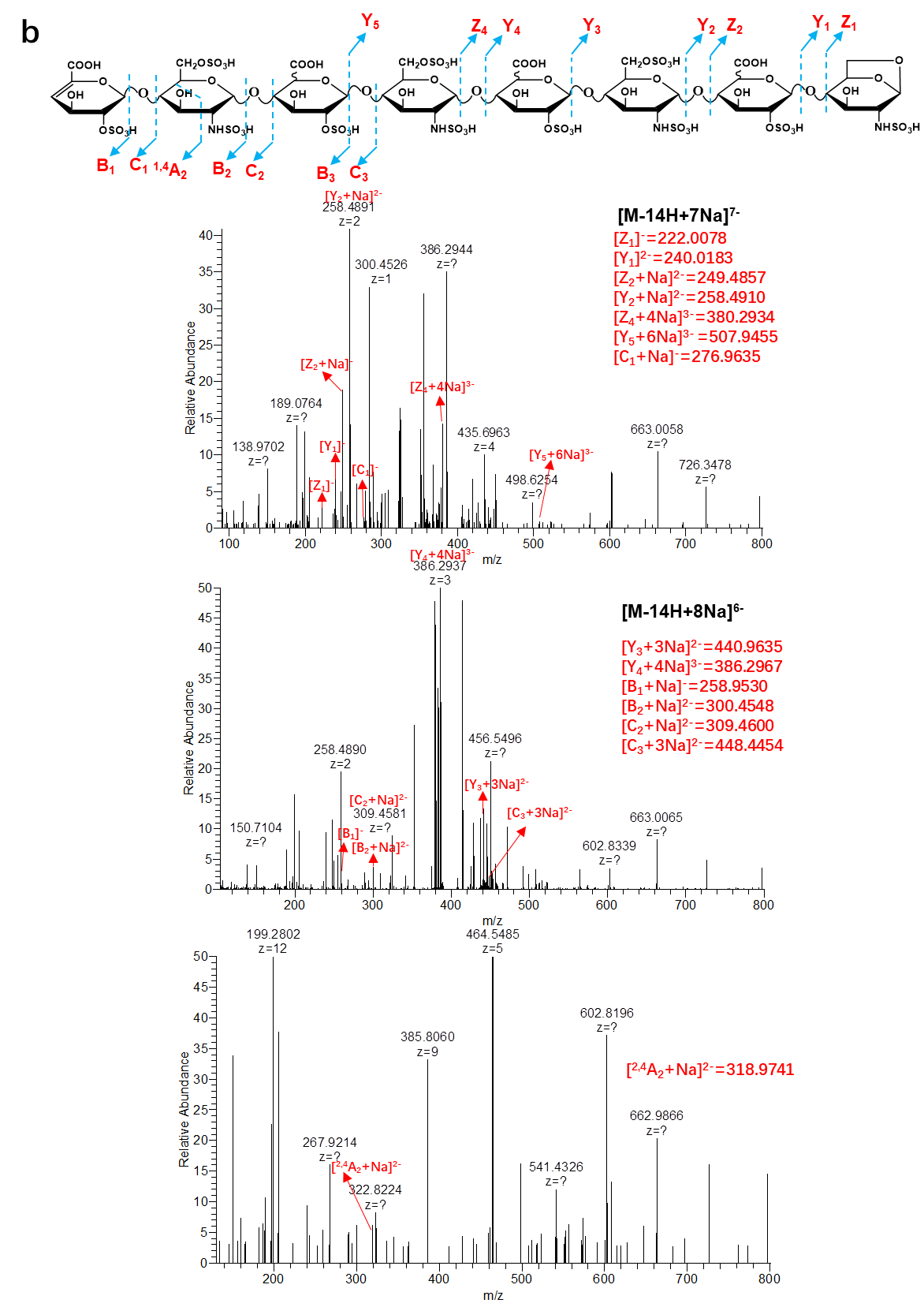


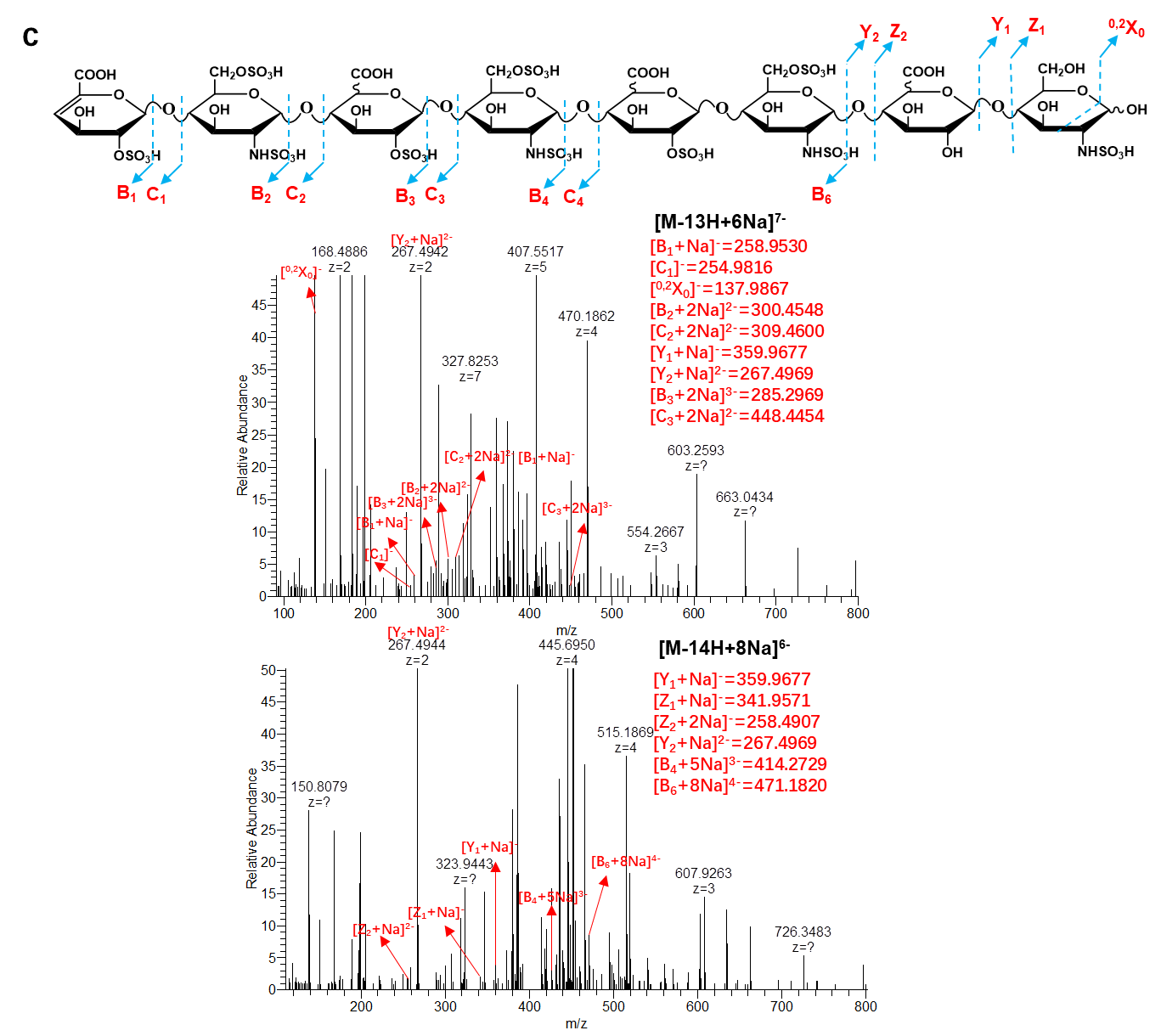


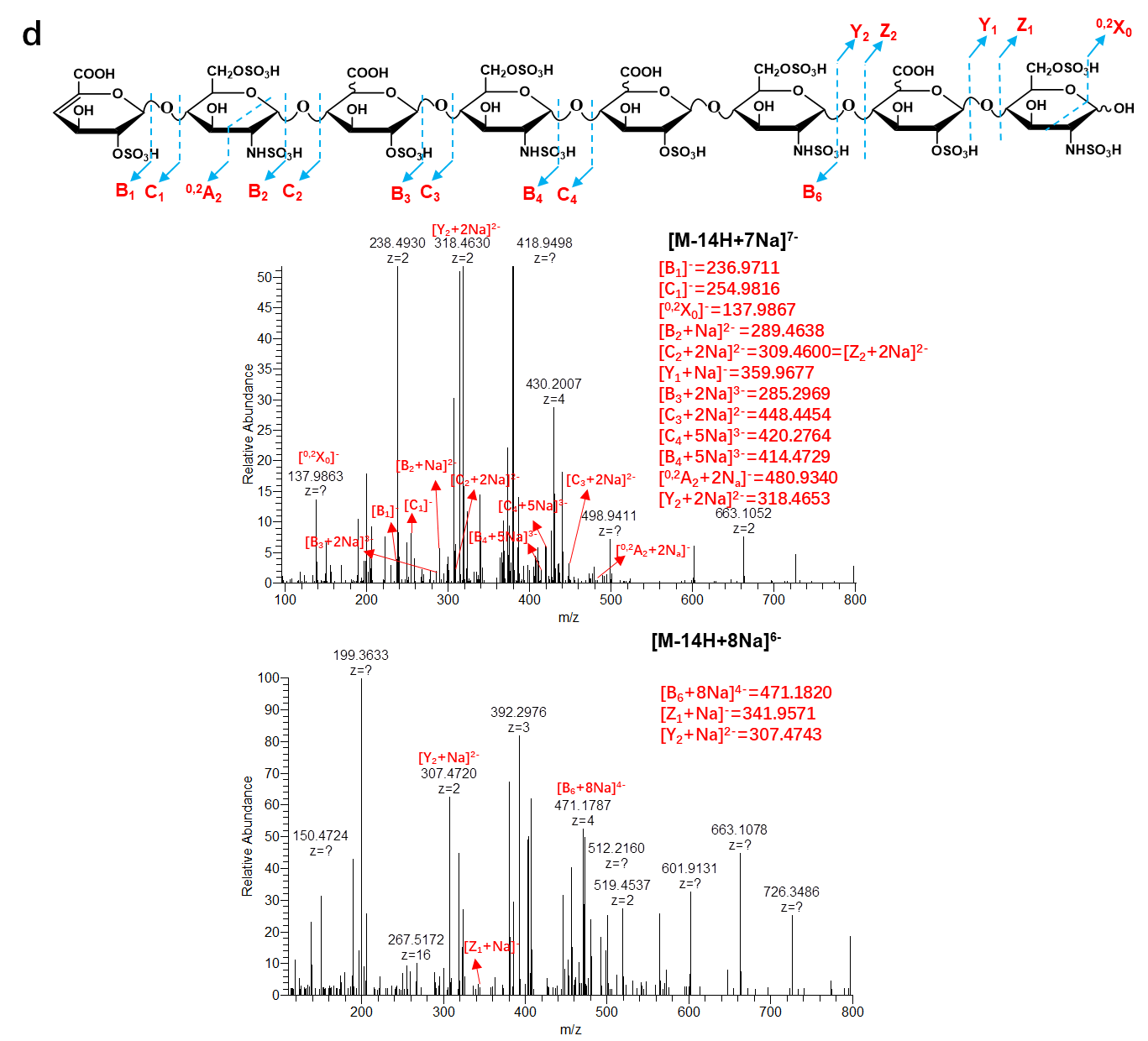


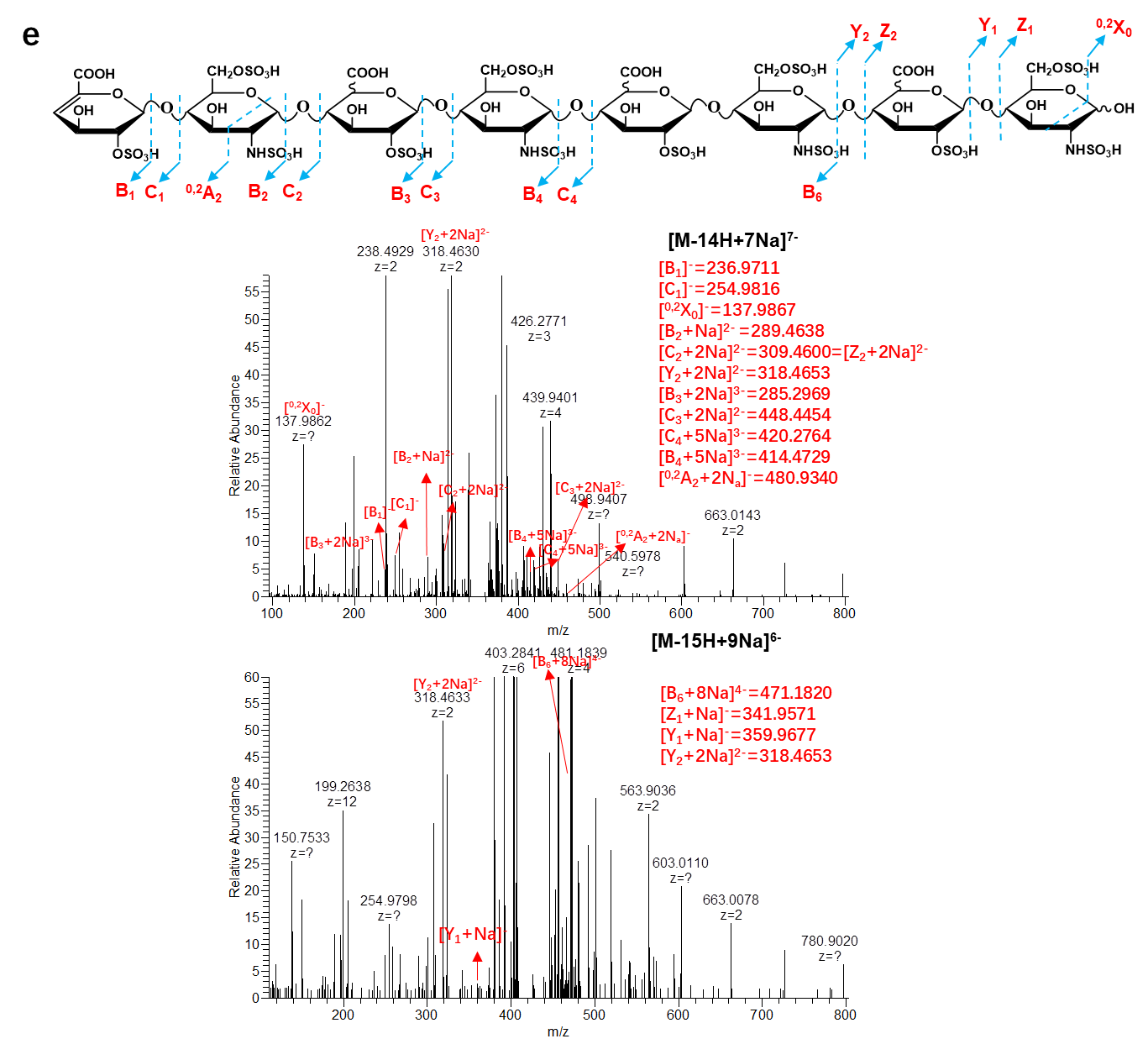


**Figure S4.** MS/MS spectra of affinity-dp8. (a) P1. (b) P2 (c) P3. (d) P4. (e) P5

**Table S1**. Information on differentially expressed proteins in label-free proteomics quantification

| **No.** | **Up-regulated** | | | | **No.** | **down-regulated** | | | |
| --- | --- | --- | --- | --- | --- | --- | --- | --- | --- |
|  | **Accession** | **Gene Name** | **Abundance Ratio** | |  | **Accession** | **Gene Name** | **Abundance Ratio** | |
|  |  |  | **Normal/DN** | **LMWH/DN** |  |  |  | **Normal/DN** | **LMWH/DN** |
| 1 | P12710 | Fabp1 | 2.578 | 2.277 | 1 | Q9JIL4 | Pdzk1 | 0.507 | 0.165 |
| 2 | Q3UNF3 | Acox2 | 2.553 | 2.548 | 2 | Q9D8C9 | Pnp2 | 0.162 | 0.057 |
| 3 | Q8N7N8 | Hmgcs2 | 6.157 | 5.064 | 3 | A0A0R4J0I1 | Serpina3k | 0.654 | 0.375 |
| 4 | Q4FJT5 | Pltp | 1.505 | 3.401 | 4 | Q03517 | Scg2 | 0.115 | 0.227 |
| 5 | Q3TUU3 | Acaa1b | 1.595 | 2.091 | 5 | Q3TN93 | Ubqln1 | 0.381 | 0.322 |
| 6 | P28825 | Mep1a | 7.653 | 2.104 | 6 | P16014 | Chgb | 0.256 | 0.192 |
| 7 | Q9NYQ2 | Hao2 | 2.432 | 2.279 | 7 | P26339 | Chga | 0.548 | 0.222 |
| 8 | Q61847 | Mep1b | 8.008 | 2.544 | 8 | Q9Z2U1 | Psma5 | 0.015 | 0.01 |
| 9 | A2AQ51 | Slc12a1 | 5.661 | 2.827 | 9 | Q14BH8 | Cacna2d1 | 0.95 | 0.272 |
| 10 | A0A4E9G461 | IGHG2 | 7.469 | 2.176 | 10 | P11983 | Tcp1 | 0.257 | 0.476 |
| 11 | Q8VEH3 | Arl8a | 2.96 | 2.087 | 11 | P24529 | Th | 0.044 | 0.164 |
| 12 | Q9CQW2 | Arl8b | 2.502 | 2.266 | 12 | Q5SVP3 | PSME2b | 0.23 | 0.457 |
| 13 | A0A0G2JFV5 | Lrrfip2 | 2.43 | 2.172 | 13 | P00920 | Ca2 | 0.195 | 0.371 |
| 14 | Q8BUM1 | Tardbp | 2.025 | 3.536 | 14 | Q50HX0 | Rab14 | 0.711 | 0.01 |
| 15 | P49429 | Hpd | 3.083 | 6.525 | 15 | Q5FWK3 | Arhgap1 | 0.216 | 0.422 |
| 16 | P26516 | Psmd7 | 3.511 | 4.792 | 16 | Q00724 | Rbp4 | 0.237 | 0.451 |
| 17 | Q3U5H8 | Hmox1 | 2.237 | 4.239 | 17 | Q91XV3 | Basp1 | 0.527 | 0.401 |
| 18 | Q8BUG2 | Cndp1 | 3.359 | 3.693 | 18 | Q545I1 | Reg3g | 0.054 | 0.098 |
| 19 | O70400 | Pdlim1 | 2.252 | 2.474 | 19 | A1L0V4 | Hist1h3i | 0.384 | 0.322 |
| 20 | S4R239 | Sftpb | 3.735 | 6.822 | 20 | Q542X9 | Sod3 | 2.243 | 0.458 |
| 21 | Q9CT19 | Hmgb2 | 2.818 | 3.722 | 21 | Q3U9U3 | Tubb6 | 0.01 | 0.01 |
| 22 | P32848 | Pvalb | 4.861 | 27.69 | 22 | Q58E35 | Rplp1 | 1.151 | 0.492 |
| 23 | Q792Y6 | Prss2 | 3.319 | 33.68 | 23 | Q3UJ92 | Cyp21a1 | 0.055 | 0.097 |
| 24 | F7C1U0 | Kctd17 | 6.586 | 9.194 | 24 | A0A1L1SU91 | Fdx1 | 0.835 | 0.45 |
| 25 | A0A140LHU0 | Adam9 | 3.005 | 5.469 | 25 | P47877 | Igfbp2 | 0.901 | 0.435 |
| 26 | Q3TKJ1 | Bcat1 | 33.446 | 7.255 | 26 | Q9D1M0 | Sec13 | 0.571 | 0.095 |
| 27 | Q9JM62 | Reep6 | 2.834 | 3.25 | 27 | Q9Z1T2 | Thbs4 | 0.526 | 0.434 |
| 28 | E9Q8X9 | Afap1 | 2.205 | 3.308 | 28 | Q9DCC4 | Pycr3 | 0.422 | 0.341 |
| 29 | P49813 | Tmod1 | 8.613 | 4.671 | 29 | F8VPU2 | Farp1 | 0.364 | 0.124 |
| 30 | Q9Z307 | Kcnj16 | 10.017 | 19.661 | 30 | P01898 | H2-Q10 | 0.07 | 0.28 |
| 31 | Q0P6I6 | Ccdc91 | 26.982 | 25.338 | 31 | Q9QZ82 | Cyp11a1 | 0.206 | 0.119 |
| 32 | Q3UNQ4 | Pafah2 | 12.927 | 11.187 | 32 | Q9DCT8 | Crip2 | 0.44 | 0.473 |
| 33 | H3BIX0 | Tcof1 | 7.158 | 5.171 | 33 | P24815 | Hsd3b1 | 0.12 | 0.218 |
| 34 | Q9DC70 | Ndufs7 | 2.748 | 4.126 | 34 | Q9DAU1 | Cnpy3 | 0.372 | 0.31 |
| 35 | A0A0R4J1R7 | Pcbd2 | 2.304 | 5.083 | 35 | O70274 | Ptp4a2 | 0.314 | 0.19 |
| 36 | O35405 | Pld3 | 5.814 | 7.046 | 36 | B1AQD4 | Rab34 | 0.106 | 0.313 |
| 37 | Q91X95 | Gna11 | 7.789 | 8.705 | 37 | Q9CQB5 | Cisd2 | 1.221 | 0.346 |
| 38 | A0A023J5X8 | ND2 | 6.523 | 4.379 | 38 | Q9U410 | UniProtKB unreviewed | 0.085 | 0.362 |
| 39 | E9PZG9 | Snrpe | 2.06 | 3.006 | 39 | D3Z0V7 | Tsc22d1 | 1.899 | 0.366 |
| 40 | Q9D846 | Ndufc2 | 4.384 | 2.656 | 40 | Q9CYA0 | Creld2 | 1.434 | 0.418 |
| 41 | A0A1B0GS44 | Tmem192 | 13.356 | 14.386 | 41 | P01629 | UniProtKB unreviewed | 0.104 | 0.234 |
| 42 | Q7TN25 | Sf3a2 | 4.385 | 4.09 | 42 | Q80VP1 | Epn1 | 2.026 | 0.32 |
| 43 | P32067 | Ssb | 6.573 | 5.41 | 43 | Q3UEW2 | Dmtn | 0.322 | 0.12 |
| 44 | Q8BMD8 | Slc25a24 | 83.207 | 15.168 | 44 | P70445 | Eif4ebp2 | 0.087 | 0.273 |
| 45 | A0A0J9YUH4 | Fryl | 21.907 | 14.729 | 45 | Q6P5G6 | Ubxn7 | 1.399 | 0.01 |
| 46 | Q8BSH0 | Atrx | 4.905 | 4.353 | 46 | P61028 | Rab8b | 0.16 | 0.337 |
| 47 | Q91XX7 | Pcdhgb2 | 3.891 | 4.061 | 47 | A2AKK6 | Acnat1 | 1.753 | 0.289 |
| 48 | Q3TN07 | Vps4b | 38.412 | 57.909 | 48 | Q3UYC1 | Mvd | 1.302 | 0.296 |
| 49 | Q3UM16 | Loxl3 | 43.588 | 4.791 | 49 | Q3ULN6 | Sccpdh | 0.75 | 0.336 |
| 50 | A0A125R9I3 | Sting1 | 3.781 | 14.165 | 50 | Q8K3K8 | Optn | 1.753 | 0.233 |
| 51 | Q9CQZ5 | Ndufa6 | 5.993 | 3.77 | 51 | Q9WTK5 | Nfkb2 | 0.01 | 0.081 |
| 52 | Q00547 | Hmmr | 4.551 | 7.577 | 52 | Q9D8C4 | Ifi35 | 0.073 | 0.203 |
| 53 | P50637 | Tspo | 6.144 | 5.278 | 53 | Q8C715 | Oxr1 | 0.959 | 0.242 |
| 54 | A0A0A6YXQ5 | Schip1 | 2.428 | 5.828 | 54 | Q8C2E6 | Slc16a1 | 0.801 | 0.368 |
| 55 | Q5SX39 | Myh4 | 1.102 | 3.021 | 55 | Q9D975 | Srxn1 | 0.052 | 0.226 |
| 56 | Q5SX40 | Myh1 | 1.471 | 7.629 | 56 | Q61390 | Cct6b | 0.347 | 0.084 |
| 57 | G3UW82 | Myh2 | 0.01 | 5.788 | 57 | Q0VBD0 | Itgb8 | 0.915 | 0.01 |
| 58 | Q91VB8 | Hba-a1 | 0.966 | 4.214 | 58 | Q9D113 | Dnlz | 2.049 | 0.277 |
| 59 | Q545G1 | Mylpf | 0.437 | 4.942 | 59 | K7TGP7 | UniProtKB unreviewed | 0.183 | 0.165 |
| 60 | Q9QWL7 | Krt17 | 1.126 | 2.254 | 60 | P52800 | Efnb2 | 0.011 | 0.085 |
| 61 | Q9EQK5 | Mvp | 0.466 | 2.28 | 61 | Q6DI81 | Nfia | 1.084 | 0.131 |
| 62 | Q91X17 | Umod | 1.946 | 2.597 | 62 | Q99LJ1 | Fuca1 | UniProtKB unreviewed | 0.107 |
| 63 | Q545T7 | Myl1 | 1.197 | 2.168 | 63 | A0A1B0GT75 | Chmp2a | 0.436 | 0.06 |
| 64 | Q9D826 | Pipox | 1.086 | 2.084 | 64 | O35522 | Psmb9 | 0.01 | 0.062 |
| 65 | Q9CR35 | Ctrb1 | 0.043 | 2.554 | 65 | Q9CZ69 | Cmtm6 | 0.607 | 0.01 |
| 66 | P21550 | Eno3 | 0.621 | 2.3 | 66 | Q3U9U9 | Eif4g2 | 1.128 | 0.208 |
| 67 | A2AIM4 | Tpm2 | 1.718 | 20.31 | 67 | Q91WQ9 | Calml4 | 0.393 | 0.319 |
| 68 | Q99J29 | Scpep1 | 0.735 | 2.151 | 68 | Q3TLQ6 | Csf1 | 0.624 | 0.113 |
| 69 | O35490 | Bhmt | 1.611 | 2.751 | 69 | E9QKV6 | UniProtKB unreviewed | 1.031 | 0.01 |
| 70 | B2RS76 | Cpb1 | 1.181 | 9.865 | 70 | D6RI20 | UniProtKB unreviewed | 1.753 | 0.263 |
| 71 | Q9EQF5 | Dpys | 1.797 | 2.913 | 71 | Q3TLM9 | Ppp4r1 | 0.84 | 0.226 |
| 72 | Z4YKH8 | Tnnt3 | 1.956 | 4.339 | 72 | G3UX98 | Nsf | 0.01 | 0.01 |
| 73 | Q8VC97 | Upb1 | 1.938 | 2.945 | 73 | Q80TU0 | mKIAA0705 | 0.01 | 0.149 |
| 74 | E9Q4P0 | Kxd1 | 0.547 | 5.967 | 74 | P09813 | Apoa2 | 0.033 | 0.367 |
| 75 | P70691 | Ugt1a2 | 0.01 | 4.746 | 75 | D3YWY5 | Aamdc | 1.992 | 0.134 |
| 76 | Q4FJZ4 | Wars1 | 1.942 | 2.475 | 76 | B0QZN5 | Vamp2 | 0.565 | 0.342 |
| 77 | Q3THQ0 | Hexa | 0.405 | 2.136 | 77 | Q3UY05 | Ndufs8 | 1.409 | 0.263 |
| 78 | Q78P93 | Asah1 | 1.68 | 2.966 | 78 | A0A075B5L7 | Igkv4-80 | 0.01 | 0.01 |
| 79 | Q9QUK9 | Try5 | 1.821 | 2.22 | 79 | Q9WV35 | Apobec2 | 0.952 | 0.262 |
| 80 | Q61704 | Itih3 | 0.556 | 2.027 | 80 | H3BJ02 | Stx5a | 1.118 | 0.01 |
| 81 | Q9CQ52 | Cela3b | 0.175 | 9.23 | 81 | Q9CQR7 | Psenen | 0.029 | 0.115 |
| 82 | A0A4E9FU10 | IGHG4 | 1.64 | 2.974 | 82 | A2A702 | Eif3m | 1.14 | 0.038 |
| 83 | P20801 | Tnnc2 | 0.486 | 7.413 | 83 | Q9JI78 | Ngly1 | 0.754 | 0.27 |
| 84 | Q6P8J7 | Ckmt2 | 1.005 | 23.939 | 84 | D6RHR9 | Dnajb12 | 0.048 | 0.209 |
| 85 | Q8C2Q8 | Atp5f1c | 0.909 | 3.37 | 85 | Q9CPT5 | Nop16 | 0.01 | 0.041 |
| 86 | P14069 | S100a6 | 0.254 | 2.054 | 86 | D3Z1C4 | Steap2 | 2.236 | 0.147 |
| 87 | E9PWK1 | Ephx1 | 0.172 | 2.252 | 87 | B1AV66 | Yipf6 | 0.501 | 0.154 |
| 88 | D3Z6P0 | Pdia2 | 1.58 | 14.033 | 88 | Q9D6X5 | Slc52a3 | 3.005 | 0.042 |
| 89 | Q8BT60 | Cpne3 | 0.743 | 2.435 | 89 | F6RDS0 | Ubr3 | 0.011 | 0.05 |
| 90 | A2A6K0 | Tnni2 | 0.497 | 3.097 | 90 | A2A7S8 | Kiaa1522 | 0.551 | 0.249 |
| 91 | Q9QWK4 | Cd5l | 0.248 | 2.32 | 91 | P35831 | Ptpn12 | 0.81 | 0.118 |
| 92 | B2RPS1 | Rab5b | 1.173 | 2.041 | 92 | Q3TD41 | Kat7 | 3.636 | 0.16 |
| 93 | Q8VDQ1 | Ptgr2 | 1.843 | 3.673 | 93 | P35918 | Kdr | 2.512 | 0.01 |
| 94 | Q9ERG2 | Strn3 | 1.61 | 3.344 | 94 | P23188 | Furin | 0.01 | 0.241 |
| 95 | Q6P8U6 | Pnlip | 0.451 | 17.551 | 95 | Q9Z0F4 | Cib1 | 0.08 | 0.147 |
| 96 | Q3TJD4 | Atp5pb | 1.718 | 5.776 | 96 | A2ICR0 | Phex | 0.203 | 0.139 |
| 97 | P62746 | Rhob | 1.337 | 4.964 | 97 | Q9MD77 | mt-Nd4l | 1.208 | 0.133 |
| 98 | Q8BG07 | Pld4 | 0.266 | 2.639 | 98 | A0A0A6YW58 | Ighv8-2 | 0.888 | 0.423 |
| 99 | O08691 | Arg2 | 1.935 | 3.481 | 99 | Q3URN5 | Aatf | 0.01 | 0.047 |
| 100 | Q61335 | Bcap31 | 1.64 | 2.208 | 100 | Q7TR66 | Olfr1083 | 0.049 | 0.149 |
| 101 | Q3TC14 | Impa2 | 1.283 | 2.958 | 101 | Q8VHH7 | Adcy3 | 0.307 | 0.084 |
| 102 | P04247 | Mb | 0.46 | 7.609 | 102 | Q60838 | Dvl2 | 0.536 | 0.163 |
| 103 | Q8K2N7 | Psmd11 | 0.938 | 3.159 | 103 | Q4KUS2 | Unc13a | 0.398 | 0.089 |
| 104 | A0A140T8N5 | Igkv6-23 | 0.066 | 2.835 |  |  |  |  |  |
| 105 | S4R225 | Wdr13 | 1.54 | 3.829 |  |  |  |  |  |
| 106 | B9EIY6 | Orm3 | 0.541 | 2.324 |  |  |  |  |  |
| 107 | B1AZ46 | Baiap2 | 1.423 | 2.802 |  |  |  |  |  |
| 108 | A0A075B5N4 | Igkv8-27 | 0.464 | 4.439 |  |  |  |  |  |
| 109 | B1AVU4 | Gm14744 | 1.302 | 2.438 |  |  |  |  |  |
| 110 | A0A075B5Y2 | Ighv1-75 | 0.01 | 8.34 |  |  |  |  |  |
| 111 | P62342 | Selenot | 0.76 | 6.487 |  |  |  |  |  |
| 112 | Q91VH6 | Memo1 | 0.443 | 3.449 |  |  |  |  |  |
| 113 | E0CZ04 | Ggact | 1.962 | 2.798 |  |  |  |  |  |
| 114 | A0A1L1SUX8 | Thy1 | 1.005 | 2.585 |  |  |  |  |  |
| 115 | Q9DD20 | Tmt1b | 1.247 | 4.393 |  |  |  |  |  |
| 116 | Q3THW5 | H2az2 | 0.283 | 3.934 |  |  |  |  |  |
| 117 | Q80VJ2 | Sra1 | 0.619 | 3.213 |  |  |  |  |  |
| 118 | Q8CC21 | Ttc19 | 1.495 | 2.27 |  |  |  |  |  |
| 119 | Q91WV0 | Dr1 | 1.333 | 5.077 |  |  |  |  |  |
| 120 | P63030 | Mpc1 | 1.549 | 4.142 |  |  |  |  |  |
| 121 | J3QP41 | Creg1 | 0.646 | 2.355 |  |  |  |  |  |
| 122 | Q6P7W2 | Shkbp1 | 1.495 | 4.921 |  |  |  |  |  |
| 123 | Q61176 | Arg1 | 1.191 | 4.537 |  |  |  |  |  |
| 124 | A0JLT5 | Mybbp1a | 0.171 | 3.137 |  |  |  |  |  |
| 125 | Q3THK3 | Gtf2f1 | 1.639 | 3.295 |  |  |  |  |  |
| 126 | E9QMV2 | Abracl | 1.121 | 3.353 |  |  |  |  |  |
| 127 | Q9QXT7 | C6 | 1.197 | 5.143 |  |  |  |  |  |
| 128 | A2ANT5 | Mup4 | 0.852 | 6.797 |  |  |  |  |  |
| 129 | Q9CPN7 | 1810009J06Rik | 0.15 | 2.51 |  |  |  |  |  |
| 130 | P63213 | Gng2 | 0.062 | 2.499 |  |  |  |  |  |
| 131 | Q3UGC1 | Strip1 | 1.127 | 3.715 |  |  |  |  |  |
| 132 | A0A075B5N2 | Igkv6-29 | 0.237 | 5.328 |  |  |  |  |  |
| 133 | A0A140T8U1 | Cyp2j7 | 0.01 | 25.286 |  |  |  |  |  |
| 134 | E9PYH2 | Acot7 | 1.861 | 4.073 |  |  |  |  |  |
| 135 | Q9JJI8 | Rpl38 | 0.01 | 18.317 |  |  |  |  |  |
| 136 | H3BK68 | Trmt1 | 1.584 | 3.554 |  |  |  |  |  |
| 137 | Q8BVU5 | Nudt9 | 0.993 | 4.121 |  |  |  |  |  |
| 138 | Z4YJD9 | Krt12 | 1.181 | 7.504 |  |  |  |  |  |
| 139 | A0A1S6GWH0 | Gabarap | 0.01 | 4.792 |  |  |  |  |  |
| 140 | Q00993 | Axl | 0.699 | 6.181 |  |  |  |  |  |
| 141 | Q9CWF6 | Bbs2 | 1.012 | 3.487 |  |  |  |  |  |
| 142 | Q8BGZ1 | Hpcal4 | 0.714 | 22.373 |  |  |  |  |  |
| 143 | Q8CIR9 | 3 | 0.334 | 4.793 |  |  |  |  |  |
| 144 | Q9JIK9 | Mrps34 | 1.86 | 4.596 |  |  |  |  |  |
| 145 | Q99MI6 | Gimap3 | 0.263 | 2.964 |  |  |  |  |  |
| 146 | U5LP42 | UniProtKB unreviewed | 0.01 | 5.453 |  |  |  |  |  |
| 147 | Q61649 | UniProtKB unreviewed | 0.09 | 2.692 |  |  |  |  |  |
| 148 | Q8BNF3 | UniProtKB unreviewed | 1.957 | 3.24 |  |  |  |  |  |
| 149 | P01631 | UniProtKB unreviewed | 0.287 | 2.141 |  |  |  |  |  |
| 150 | Q8C0Y5 | UniProtKB unreviewed | 1.212 | 3.372 |  |  |  |  |  |
| 151 | Q8C5B4 | UniProtKB unreviewed | 1.877 | 5.699 |  |  |  |  |  |
| 152 | X5J5C8 | UniProtKB unreviewed | 0.195 | 2.198 |  |  |  |  |  |
| 153 | P01626 | UniProtKB unreviewed | 0.01 | 4.917 |  |  |  |  |  |
| 154 | A0A097PUG4 | UniProtKB unreviewed | 3.099 | 2.305 |  |  |  |  |  |
| 155 | Q6LEM8 | UniProtKB unreviewed | 4.374 | 3.415 |  |  |  |  |  |
| 156 | A2NW56 | UniProtKB unreviewed | 2.222 | 3.478 |  |  |  |  |  |
| 157 | X5J5H0 | UniProtKB unreviewed | 6.53 | 9.197 |  |  |  |  |  |
| 158 | Q99LA8 | Dpep1 | 100 | 100 |  |  |  |  |  |
| 159 | Q9CPQ9 | Aldoart1 | 100 | 100 |  |  |  |  |  |
| 160 | B2RY26 | Myh7 | 100 | 100 |  |  |  |  |  |
| 161 | Q99LD4 | Gps1 | 100 | 100 |  |  |  |  |  |
| 162 | Q8BHS3 | Rbm22 | 100 | 100 |  |  |  |  |  |
| 163 | Q8BXB6 | Slco2b1 | 100 | 100 |  |  |  |  |  |
| 164 | A0A7N9VSY8 | Wdr54 | 100 | 100 |  |  |  |  |  |
| 165 | Q924A2 | Cic | 100 | 100 |  |  |  |  |  |
| 166 | Q9D6K2 | Tagln3 | 100 | 100 |  |  |  |  |  |
| 167 | Q3UWZ0 | Trim75 | 100 | 100 |  |  |  |  |  |
| 168 | Q8CCP0 | Nemf | — | 100 |  |  |  |  |  |
| 169 | Q8BFZ3 | Actbl2 | — | 100 |  |  |  |  |  |

**Table S2.** Primer for the synthesis and degradation enzymes of HS

| **Name** |  | **Sequence（5’-3’）** | **Size** |
| --- | --- | --- | --- |
| Homo GAPDH | Forward | TCAAGAAGGTGGTGAAGCAGG | 115bp |
|  | Reverse | TCAAAGGTGGAGGAGTGGGT |  |
| Homo EXTL1 | Forward | CATTGATGGGCACAGGAAGG | 230bp |
|  | Reverse | ATTCGTTGGTCCTCTCAGCA |  |
| Homo EXTL2 | Forward | GATGCTCATGTTGCGTAGGG | 185bp |
|  | Reverse | CATCTGGTGCCTTCTCTCCA |  |
| Homo EXTL3 | Forward | GGTGCTGCCTTCTTTCACAA | 225bp |
|  | Reverse | CTCGTGGAAGTGGGAGTCAT |  |
| Homo EXT1 | Forward | GACTGGCAAAAGCACAAGGA | 235bp |
|  | Reverse | CTATGACGGCAGCTTGGTTC |  |
| Homo EXT2 | Forward | GGGTGTCCTTTCTGTCCGTA | 161bp |
|  | Reverse | CAATGACAACCGGGACACAG |  |
| Homo HPSE | Forward | AGCGTGCAAGGTTCAAAGAG | 197bp |
|  | Reverse | CCATGAGGTCCCAAAGGTCT |  |
